# A convenient analytic method for gel quantification using ImageJ paired with Python or R

**DOI:** 10.1371/journal.pone.0308297

**Published:** 2024-11-21

**Authors:** Cassidy Tomlinson, Ashwini Rajasekaran, Karine Brochu-Gaudreau, Claire Dubois, A. James Farmilo, Pavel Gris, Ariane Khatiz, Amanda Matthews, Marjo Piltonen, Abdelaziz Amrani, Denis Gris

**Affiliations:** 1 Faculté de Médecine et des Sciences de la Santé, Université de Sherbrooke, Sherbrooke, QC, Canada; 2 deutraMed, Collingwood, ON, Canada; Nathan S Kline Institute, UNITED STATES OF AMERICA

## Abstract

In recent years, due to the COVID-19 pandemic, there was a surge of research on mRNA therapeutics. The applications are broad and include vaccination, cancer therapy, protein replacement, and immune modulation. mRNA therapeutics have advantages over other nucleic acid therapies because of the reduced risk of mutagenesis. On the other hand, mRNA therapeutics have a large caveat due to its inherent instability, which makes it susceptible to degradation throughout all stages of production, storage, and *in vivo* application. Decades ago, agarose gel electrophoresis was developed to separate and resolve nucleic acids based on size. Since then, the evolution of image analysis tools, such as ImageJ, has facilitated semi-quantitative evaluation of concentration based on band intensity, and qualitative observation of RNA integrity from gel electrophoresis. Instruments utilizing capillary electrophoresis, like the Agilent 2100 Bioanalyzer, that use microchip linear acrylamide gel electrophoresis have been demonstrated to be superior to agarose gel electrophoresis in studying RNA quality. Due to the higher cost of usage, they are less accessible to the average lab than agarose electrophoresis. In this work, we review the fundamentals of mRNA assessment and propose a full-lane quantification (FLQ) method, which is a fast, simple, and inexpensive method to analyze RNA degradation from agarose gels using ImageJ paired with Python and R. This measures the area under the curve of the product peak, degradation zone, and a combined score to provide sensitive means to evaluate the degradation of mRNA. This method provides measures of the degradation profile within each lane comparable to an RNA integrity number from bioanalyzers. Using this cost-effective method, we demonstrate that the degradation index is a sensitive measure that reflects the degradation and preservation of mRNA patterns.

## Introduction

Since the COVID-19 pandemic, ribonucleic acid (RNA) has become a promising option for many therapeutics as an alternative to deoxyribonucleic acid (DNA) based- and protein-based approaches [1]. mRNA is the intermediate molecule between DNA and the production of proteins [1]. mRNA therapeutics are quickly broadening their scope, having applicability in infectious diseases, cancer, and genetic disorders [2, 3]. However, mRNA’s susceptibility to degradation complicates its production, quality control testing, and experimentation [4, 5]. It is inherently chemically unstable and is subject to enzymatic degradation by ubiquitous RNases and hydrolysis [1, 4, 6]. This spotlight on mRNA therapeutics has increased the demand and pressure to develop more stable and cost-effective production methods as well as convenient and reliable methods of quantification [3]. To quantify and characterize the quality of mRNA, analytical techniques such as UV spectroscopy, fluorescent-based assays, gel electrophoresis, blot analysis, chromatography, and sequencing techniques are used [2, 3]. Poveda et al provided a detailed description of each technique in 2019 [2].

Electrophoretic separation is based on the charge, size, and shape of the molecule [7]. It can be done using techniques including capillary electrophoresis, microchip electrophoresis, and supporting media like paper, film, or gels [8]. There are vertical and horizontal systems. The latter is more versatile, including methods such as zone electrophoresis, immunoelectrophoresis, affinity electrophoresis, pulse-field gel electrophoresis, and using many variations of agarose and polyacrylamide gels for proteins and nucleic acids depending on the size of the molecules [8]. Gel types include starch, dextran, agarose, and polyacrylamide [8, 9]. In all cases, the gel matrix acts as a molecular sieve. The interactions between the electrical force and the gel matrix result in different migration rates of molecules through the gel [10]. For detection and size analysis of biomolecules, vertical systems with polyacrylamide gels with smaller, more stable pores are used more often for protein and small nucleic acid separation, while horizontal systems with agarose are used for larger proteins and nucleic acids [7]. These gels can be analyzed directly or undergo further processing for blotting techniques [11].

Agarose gel electrophoresis has been recognized as a common, inexpensive, and effective method for nucleic acid separation since 1970 [8, 9]. Due to the molecular strength of agarose, the concentration can vary depending on the needed size of the pores throughout the gel, where higher concentrations are needed for smaller pores aka smaller molecules [9]. Traditional agarose gel electrophoresis’s range is from 100bp to 25kb [9]. For fragments smaller than 100bp, low melting agarose, polyacrylamide gel electrophoresis, or capillary electrophoresis is required, and for larger than 25kb, pulse field electrophoresis is needed [9]. To allow negatively charged nucleic acids to travel through the gel from the negative to the positive end, a low and constant voltage is needed, approximately 10V/cm of gel [8, 9]. Voltage influences the speed of the molecules travelling through the gel. The secondary structure or shape of the RNA molecule can interfere with the separation of the molecules in non-denaturing gels [12]. Denaturing gels can be used to account for this. Denaturants such as urea, formaldehyde, or formamide can be added to the gel [12]. These toxic and carcinogenic substances require further processing to be compatible with staining techniques and may cause a diminished signal intensity [13].

**Fig 1:**
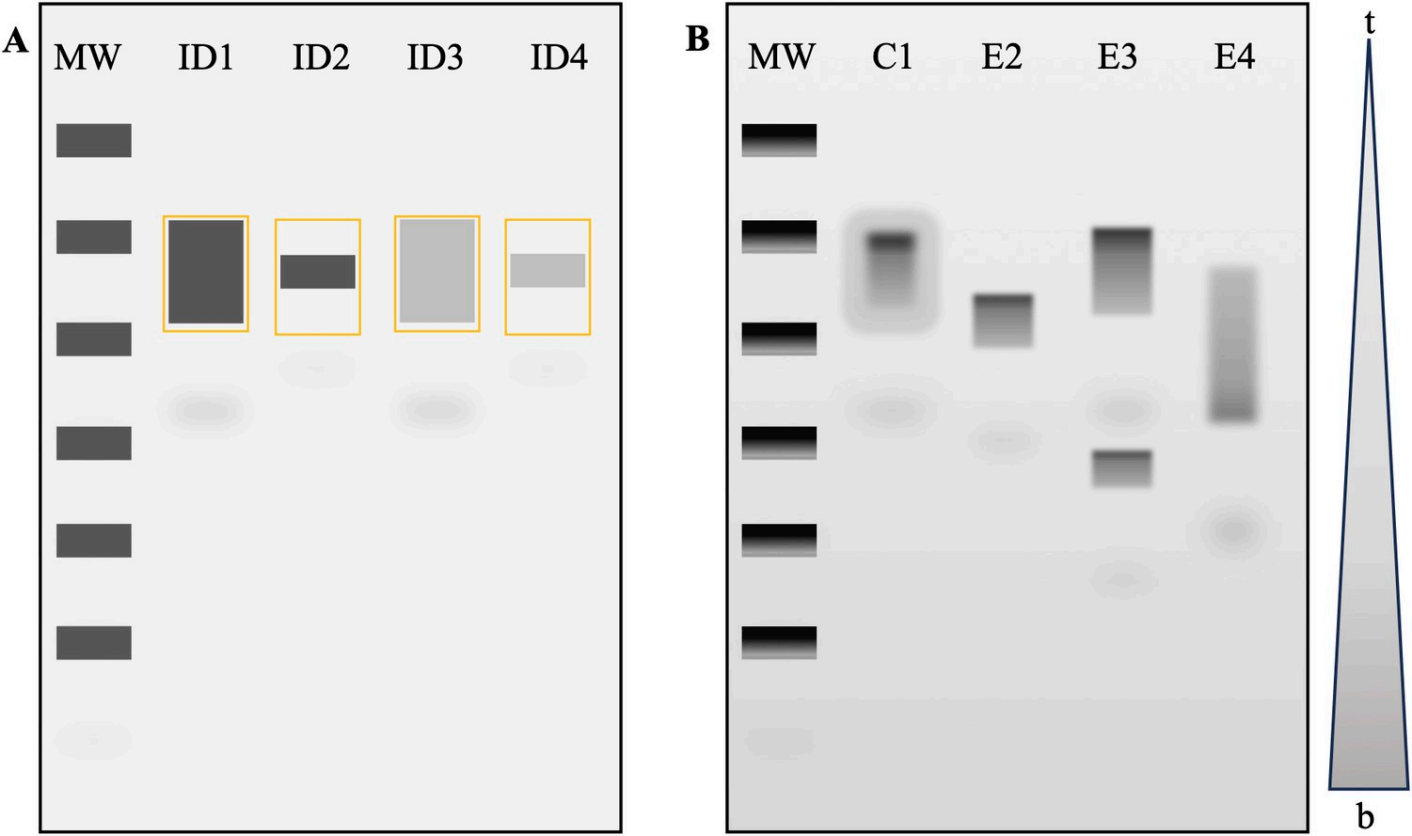
Gel quantification. **A)** Theoretical image of a typical method for quantification via ImageJ whereby bands of interest are selected and assuming ideal conditions (ID1, ID2, ID3, ID4) with an equal background. **B)** Hypothetical gel slab depicting a typical degradation experiment with a top-to-bottom gradient (t-b). The control condition C1 is compared to experimental E2, E3 and E4. Note that as the mRNA molecule degrades, the band size may decrease E2, become two bands E3, or have no clear definition E4. It can also be split in two when a certain amount of mRNA is degraded more rapidly and travels further in the gel. MW denotes the molecular weight ladder. S1 Dataset is provided as an example data set.

To observe the nucleic acids in the agarose gel there needs to be a stain added during gel preparation. Such stains include Ethidium Bromide (EtBr), SYBR Gold, SYBR green, Crystal Violet, and Methyl Blue [9]. EtBr is the most common due to its price and effectiveness [9]. Intercalating agents can bind to DNA and RNA by inserting the planar aromatic moiety between base pairs that causes structural changes to enhance fluorescence and induce functional arrest of the nucleic acid [14, 15]. EtBr intercalates in a concentration-dependent manner and indicates the number of nucleic acid fragments within a band based on band brightness [16]. EtBr has a positive charge and travels oppositely to nucleic acids [9]. Upon exposing the gel to UV in an Imaging System, the size can be determined by comparing the placement of sample bands to the bands of a DNA or RNA mixture with fragments of known sizes [9]. When molecular weight is known, the quantification of the bands on the gel is straightforward (Fig 1A). However, it is important to note that using a non-denaturing gel will reduce the precision of the molecule weight measurement due to the hydrogen bonds that introduce base pair interactions depending on the temperature and buffer used [13].

Gel electrophoresis is deemed semi-qualitative for RNA quantification and integrity evaluation and lacks a method for analysis of product degradation [16–18]. The size, shape, and quality of the bands can vary among lanes depending on the comb, agarose concentration, sample volume buffers used, and voltage [19, 20]. High voltage can cause asymmetric heating, resulting in lane and band abnormalities such as slanting, broadening, compression, or even causing a ‘smiling effect’ where the middle of the band travels faster than the outside [7]. Degraded products result in a smeared pattern down the lane; depending on the degree of degradation, this can result in a splitting or the absence of a band [20]. The resulting bands can be analyzed for size and intensity, but quantification is difficult for degradation experiments on standard agarose gels as the bands are not uniform or smeared (Fig 1B) (S1 Dataset). In non-denaturing gels, secondary structures interfere with the separation of the degraded products and thus may not represent the true molecular size/weight [12, 21]. To image gels, a gel documentation system is used. They include a dark chamber for the gel, UV fluorescent tubes or blue light transilluminator, a camera, and software to control the camera [22].

ImageJ, previously known as NIH Image, is a free biological imaging and scientific computing program that has made tremendous strides in the past few decades [23]. ImageJ offers features to support gel electrophoresis analysis [24]. The software measures each lane relative to each other so that the samples must be on the same gel for comparison, tools such as the rectangle tool and *Plot Lane* function can be used to maintain continuity between lane and gel quantifications [18, 24]. ImageJ has band band-finding feature but will not give the molecular weight without the quantification of a ladder to interpolate the base pairs associated with the distance run on the gel [25].

In 1999, a new automated technology using microfluidics, microcapillary electrophoresis and laser-induced fluorescence (LIF) for the size-based separation of DNA, RNA and proteins, Agilent 2100 Bioanalyzer, was introduced [8, 17]. Further systems such as Fragment Analyzers, TapeStation, FemotoPulse, and other automated electrophoresis systems were developed for various types of nucleic acids [26]. Requiring only routine pipetting and basic computer skills, Bioanalyzers use microchip electrophoresis, which is a miniature version of capillary electrophoresis [8, 27]. The typical glass chip has an interconnected network of fluid reservoirs and microchannels filled with gel-dye mixtures [8, 27]. RNA stained with intercalating dye is separated based on molecular weight within the microfluidic chips via electrodes, resulting in voltage-induced size separation in the gel-filled channels [17, 27]. RNA sizes are detected with LIF to provide an electropherogram that correlates fluorescence with the amount of RNA of given sizes [17]. Smaller sample sizes are required due to increased sensitivity; the samples can be quantified in 30 minutes, and integrity, purity, and quantitation can all be evaluated on a single platform [17, 27, 28].

To display data from bioanalyzers, the software develops virtual gels, electropherograms, and migration-time plots, allowing users to develop an algorithm to determine RNA Integrity Numbers (RIN) [17, 27]. To standardize RNA quality evaluation with bioanalyzers, a software algorithm and a prediction model were developed using information theory and Bayesian statistics to provide an RIN [17]. Degradation of RNA is continuous, so there are no natural stages or categories but the electropherograms can evaluate the process of degradation by visualizing electrophoretic traces as signal intensities [17]. In the study by Schroeder *et al*., the band signals decreased, and numerous small band signals appeared along the baseline, which resulted in peaks representing only degraded material, demonstrating the range of assigned RINs [17]. While bioanalyzers have certain enhancements over gel slabs, the advantages of traditional gel electrophoresis include the ability to resolve transcripts larger than 3000nt [28] and the accessibility [29].

In this paper, we will discuss a new, full-lane quantification (FLQ) method developed to optimize the quantification of mRNA degradation in the entire lane without prior knowledge of the size of the molecule. We will explain how we recreated the outputs from ImageJ with the provided-for-use algorithms using Python and R. We provide evidence for the FLQ method of analysis to obtain a preservation score for nucleic acids using *in vitro* transcribed (IVT) mRNA and compare plot profiles of the FLQ method to capillary electrophoresis instruments. Given that capillary electrophoresis instruments have a method to quantify RNA degradation using RINs via the ribosomal subunits absent from IVT-mRNA [17], we will compare our preservation scores to RINs utilizing total RNA. Gel electrophoresis, regardless of the gel type, is a common and semi-quantitative method for RNA evaluation method based on separation by molecular weight, but there is no protocol for a clear evaluation of degradation product quantitation [16].

## Methods and materials

### Bacterial transformation

Competent *E*. *coli* cells in glycerol were thawed from -80°C. In a falcon tube, 20–50 μL of cells were incubated with 10–100 ng of DNA encoding for human NLRx1 variant 4 then were transformed via heat shock in a water bath at 42°C. The transformed cells were plated and incubated at 37°C for 24 hours in LB agar containing ampicillin. 3–4 colonies were inoculated into liquid LB media with ampicillin and were left shaking at 37°C for 24 hours. The cells were separated from the LB media with 4°C centrifugation at 6000 RPM for 15 minutes. Following the GenElute Maxiprep protocol, the DNA was isolated from the cells. The concentration was read in a BioDrop analyzer.

The 10 μg of DNA was linearized using 2 μL of restriction enzyme, 10μL of cutSmart buffer and filled up to 100 μL of H_2_O. This was incubated at 37°C for 1 hour. The DNA was then purified using the QIA Prepkit and the yield was again read in the BioDrop analyser.

### *In vitro* transcription

A mixture of 1μg of linearized DNA, 2 μL of each nucleotide (A, G, C, U-TP), 2 μL of reaction buffer, 2 μL of polymerase, and 8 μL of RNase-free H_2_O was, vortexed and incubated for 2 hours at 37°C. Next, 2.5 μL of DNase, buffer and 57.5 μL of nuclease-free H_2_O were added to the tubes. RNA Purification was done following the Monarch kit protocol. The final concentration was read in the BioDrop.

### RNA extraction

Total RNA was extracted from U118mg and U87mg cell lines obtained from ATCC by following the standard protocols for QIAGEN RNeasy Kit (#74136) and TRIzol^®^ (#15596018) The concentrations were measured in a BioDrop spectrophotometer.

### Degradation

The total RNA samples were incubated at 37°C or 45°C for the allotted time points, 0 days, 2 days, 4 days, 7 days, and 10 days or 0 hours, 3 hours, 6 hours, 12 hours, 24 hours, 36 hours, and 48 hours.

The IVT mRNA samples were incubated at 37°C for the allotted time points, 0 days, 2 days, 4 days, 7 days, and 10 days.

### Agarose gel electrophoresis

Gel electrophoresis was performed using 2% agarose in a buffer containing 1xTris, acetate, and ethylenediaminetetraacetic acid (EDTA) (TAE) and 5 μL EtBr. The gel electrophoresis was conducted at 96V for ~45 minutes. Digital images were acquired with BioRad GelDoc.

### Analysis with ImageJ

1. To open an image *File>open*.
2. Turn the image black and white *Image> type>16 bit* (dark background and light bands).
3. To acquire the entire lane signal, the gel needs to be rotated 90 degrees; *image>transform>rotate 90° right*.
4. Select the rectangle tool and draw a box around the first lane (typically the molecular weight ladder) so that the box includes the entire lane length.
5. To show the graph, click *Analyze>Plot profile*.
6. To get data click *Live>List*. Only click Live for the first lane.
7. Copy the whole list into an Excel sheet and close the list.
8. Hover the cursor over the box outline until the pointer appears and click. Use arrow keys to move the box to the next lane, do not use the left or right arrows.
9. Repeat steps 6–8. Maintain the box and measure a blank lane; if all lanes are used, the box can be narrowed to measure 3–5 spaces between lanes (box width will need to be adjusted). Alternatively, the background can be computed using top and the bottom grey values of the gel.
10. Ensure that the Excel sheet has columns in the following order: distance, background (if measuring directly), nucleic acid ladder, control, and each time point. then add any experimental conditions such as solvent, transcript differences, temperature, etc. Include the gel name/number and date in the file name.

### Codes

The script for Python was developed in the environment Spyder. The script for R was written in RStudio. The corresponding script to the desired programming software must be used. The data file pathway must be changed to match the desired Excel file. The indicated lines of code must be modified to match the user’s own data sets (number of columns/rows, column names, etc).

The protocol described in this peer-reviewed article is published on protocols.io.

## Results

**Fig 2:**
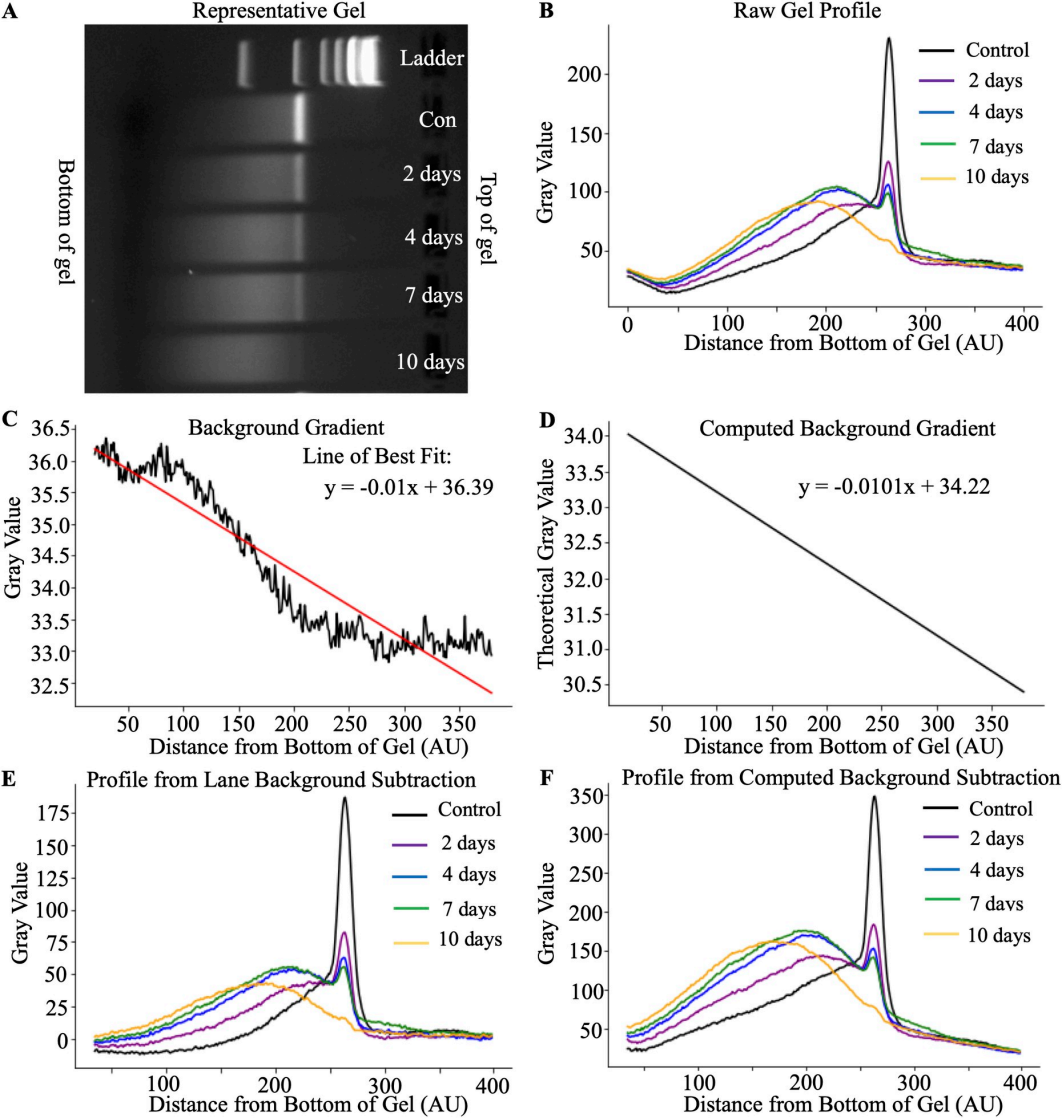
Background evaluation of IVT-mRNA. **A)** Representative image of the gel vertical background. Note that the gel was rotated to show the background horizontally to graphical representations of the signal in the gel **(B)**. The Y-axis represents the gray value, and the X-axis represents the indexed distance from the bottom of the gel. **C)** Raw background signal in the blank lane. **D)** Computed background gradient based on the signal at the top and bottom of the gel in the control lane. In C and D, the straight line depicts linear regression of the decline of the signal. **E) and F)** show signal after subtraction of the background using raw and computed signal, respectively.

Using a representative gel shown in Fig 2A, IVT-mRNA for the human NLRx1 variant 4 at various degradation points was loaded into 2% agarose gels. The backgrounds of agarose gels are not uniform and follow a linear gradient along the horizontal axis, as shown in Fig 2B. The backgrounds of agarose gels are not uniform and follow a linear gradient along the horizontal axis, as shown in Fig 2B. Note that the gel was rotated 90 degrees. Since the signal comparison is performed using the entire lane, the background must be subtracted along the entire length of the lane. In the signal extracted from the representative gel, the intensity of the background is higher at the top (400 AU) and lower at the bottom (0 AU) of the gel (Fig 2B). The background gradient can be acquired by measuring a blank lane or averaging at least three inter-lane spaces (Fig 2C). Alternatively, the gradient can be computed using the difference between the gray values of the initial and final sections of the gels of a control lane. Then, the background can be subtracted from each lane based on the index position (Fig 2D). Both methods demonstrated a similar downward trend of background along the gel.

By subtracting the background gradient from each lane, the grey values of lane intensity profiles were equalized between the top and bottom of the gel (Fig 2E and 2F). The control sample, by definition, has the lowest degradation; it has the tallest and narrowest peak. Therefore, it was used as the reference point for all the experimental conditions. As degradation progressed, the intensity of the peaks decreased, while broader signals were formed closer to the bottom of the gel, indicating increases in the smaller fragments that had travelled further through the agarose gel.

**Fig 3:**
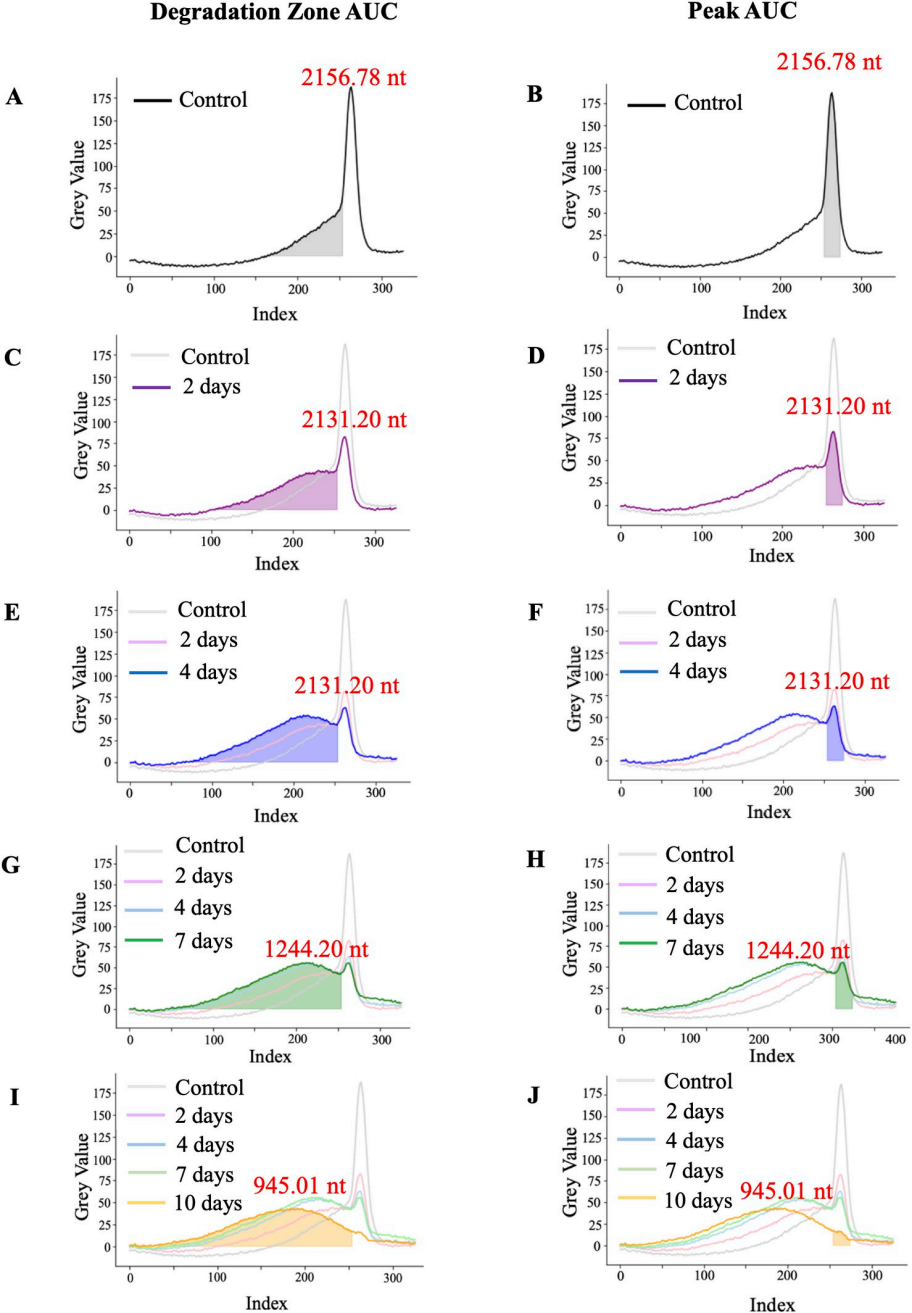
Signal intensity of IVT-mRNA bands. Assessment of degradation zone of mRNA after IVT-control **(A)** and incubation at 37 degrees for 2 days **(C)**, 4 days **(E)**, 7 days **(G)**, and 10 days **(I)**. Assessment of the amount of original product in mRNA after IVT-control **(B)** and incubation at 37 degrees for 2 days **(D)**, 4 days **(F)**, 7 days **(H)**, and 10 days **(J)**. The evaluated signals are depicted by filled areas; the lines are provided for relative comparison to other samples. In each sample, the size of the maximal value is denoted beside the peak.

Once the background was subtracted, we proceeded to estimate the amount of IVT-mRNA product in each lane using the area under the curve (AUC). The peak curve includes the 20 indexes that surround the maximum gray value, indicating the size of most of the product. The degradation curve includes the rest of the signal to the left of the peak curve. Fig 3 depicts the degradation pattern evolving during the 10 days of the experiment. Panels A, C, E, G, and I in Fig 3 highlight the appearance of smaller molecular weight products denoted as the degradation zone. Panels B, D, F, H, and J highlight the decrease in the AUC of the peak. The development of additional peaks as the mRNA degrades is visible in the degradation zone. The peak begins to broaden as there are various size fragments, and the signal shifts leftward as the smaller molecules travel further in the gel. Note the reciprocal relationship between the AUC of the peak of the main product and the AUC of smaller fragments.

**Fig 4:**
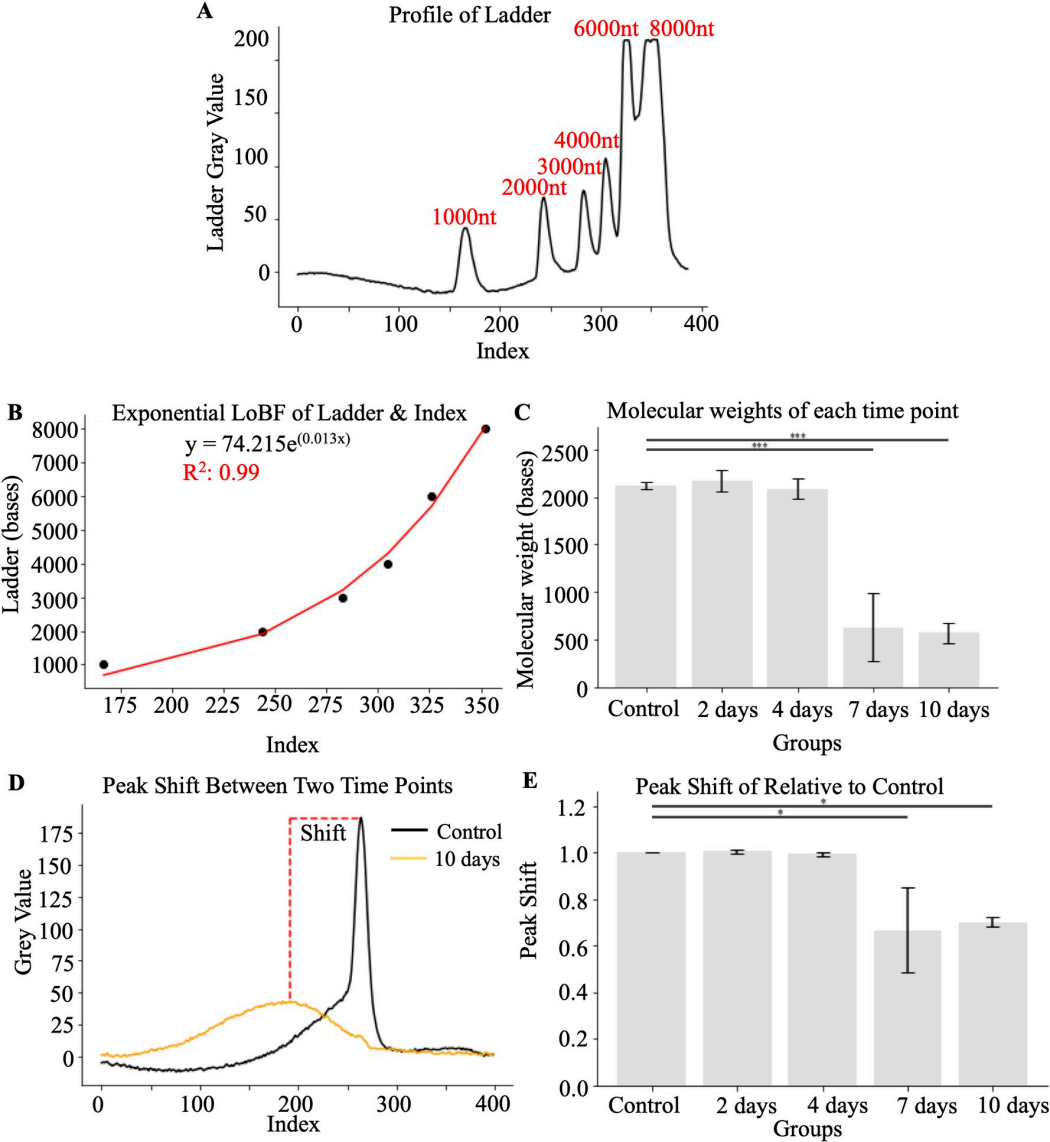
Quantification of the degradation of the IVT-mRNA product. **A)** Representative profile of ladder using lane background subtraction with manufacturer-defined molecular weight. **B)** Show the best-fit exponential curve and formula for molecular weight assessment based on the position of the gel. **C)** Decreases in maximal values of molecular weight of mRNA after various times of incubation at 37 degrees. **D)** Peak shift defined by the difference of maximal molecular weight values after incubations. **E)** Quantification of maximal values differences. *, p<0.05, **, p<0.01, ***, p<0.001 determined by ANOVA followed by Tukey test (N = 3).

To evaluate the molecular weight of the resulting products, we measured the index position of each of the molecular weight bands (Fig 4A), which corresponded to the exponential function y = 74.215e^0.013x^ with R^2^ = 0.99 (Fig 4B). This helped to define the declined molecular weight of the prevalent product (Fig 4C). Based on the maximum peaks of each time point, the determined size of the highest quantity of undenatured fragments for the control, 2, 4, 7 and 10 days were 2118.90 nt ± 21.98, 2171.19 nt ± 65.27.40, 2087.07 nt ± 59.81, 630.90 nt ± 205.49 and 569.40 nt ± 60.95. Alternatively, the degree of degradation can be expressed as a peak shift (Fig 4D), a ratio of change of molecular weight relative to control values (Fig 4E). Note that the disappearance of the initial peak values of the original product does not become obvious until 7 days of incubation at 37°C.

**Fig 5:**
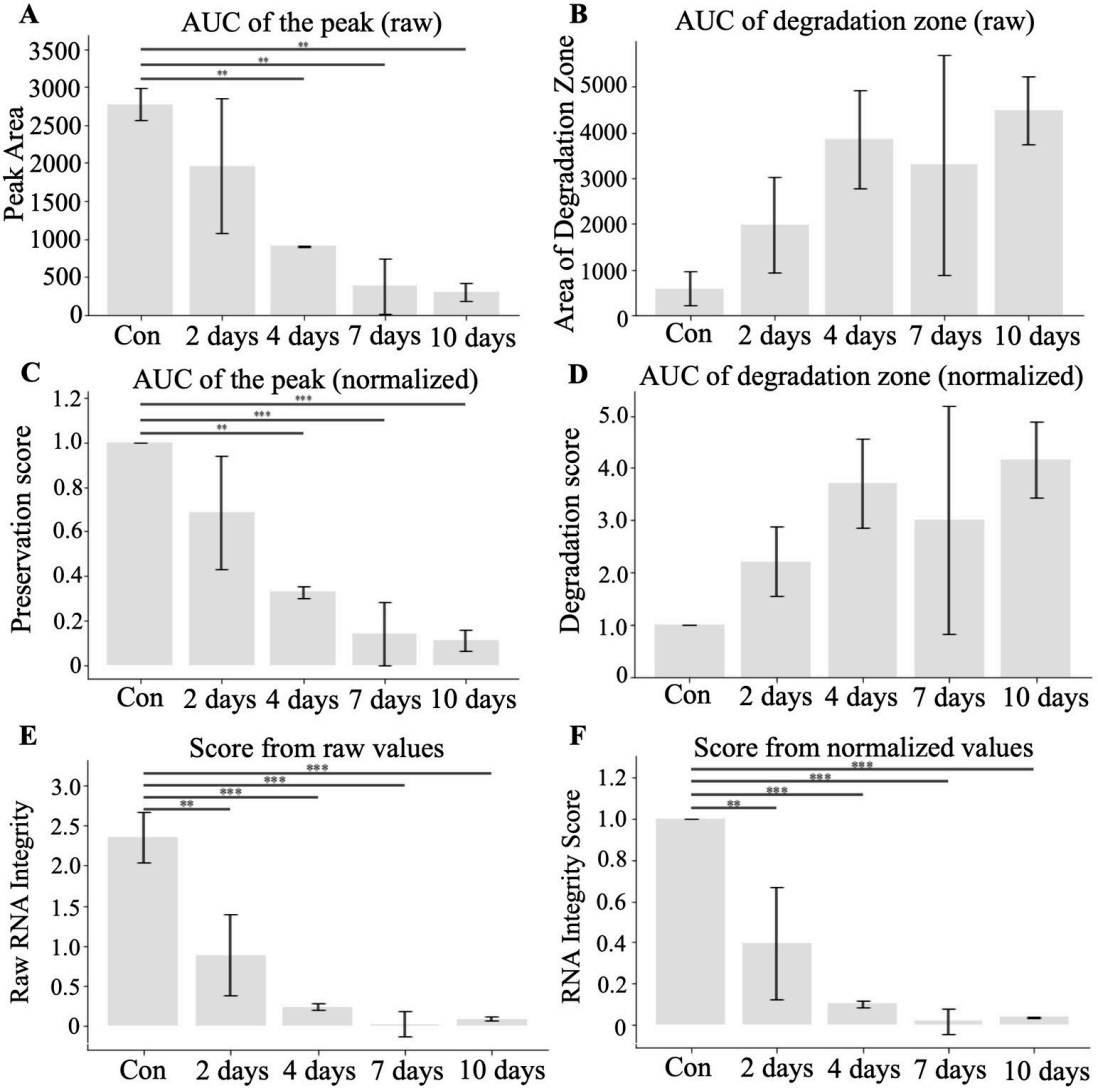
Quantification of the signal degradation using AUC. **A)** AUC of the peak zone comparisons. **B)** Degradation zone AUC. **C)** AUC values of the peak zone and **D)** degradation zone normalized to control to represent fold change. Preservation scores that incorporated both AUC the peak and AUC of the degradation zones either using raw **(E)** or normalized to control **(F)** values. *, p<0.05, **, p<0.01, ***, p<0.001 determined by ANOVA followed by Tukey test (N = 3).

To measure the degree of degradation of mRNA during the experiment we quantified the AUC of the peak and degradation curves. Fig 5A shows significant decreases in the AUC of the peak at 4, 7, and 10 days compared to the control. The degradation zone AUCs, although increased, did not reach statistical significance at later time points (Fig 5B) due to increased variability of degradation. Normalization using controls reduced variability shown in panels C & D. Since degradation of the mRNA constitutes a reduction of the molecular weight of the original product and increases in degradation fragments, we combined these two metrics to compute the preservation score. The preservation scores were more reflective of the degradation dynamics of mRNA. Panels E and F in Fig 5 show raw and normalized scores, respectively. There were significant decreases compared to control in each time point during incubation, including 2, 4, 7, and 10 days.

**Fig 6:**
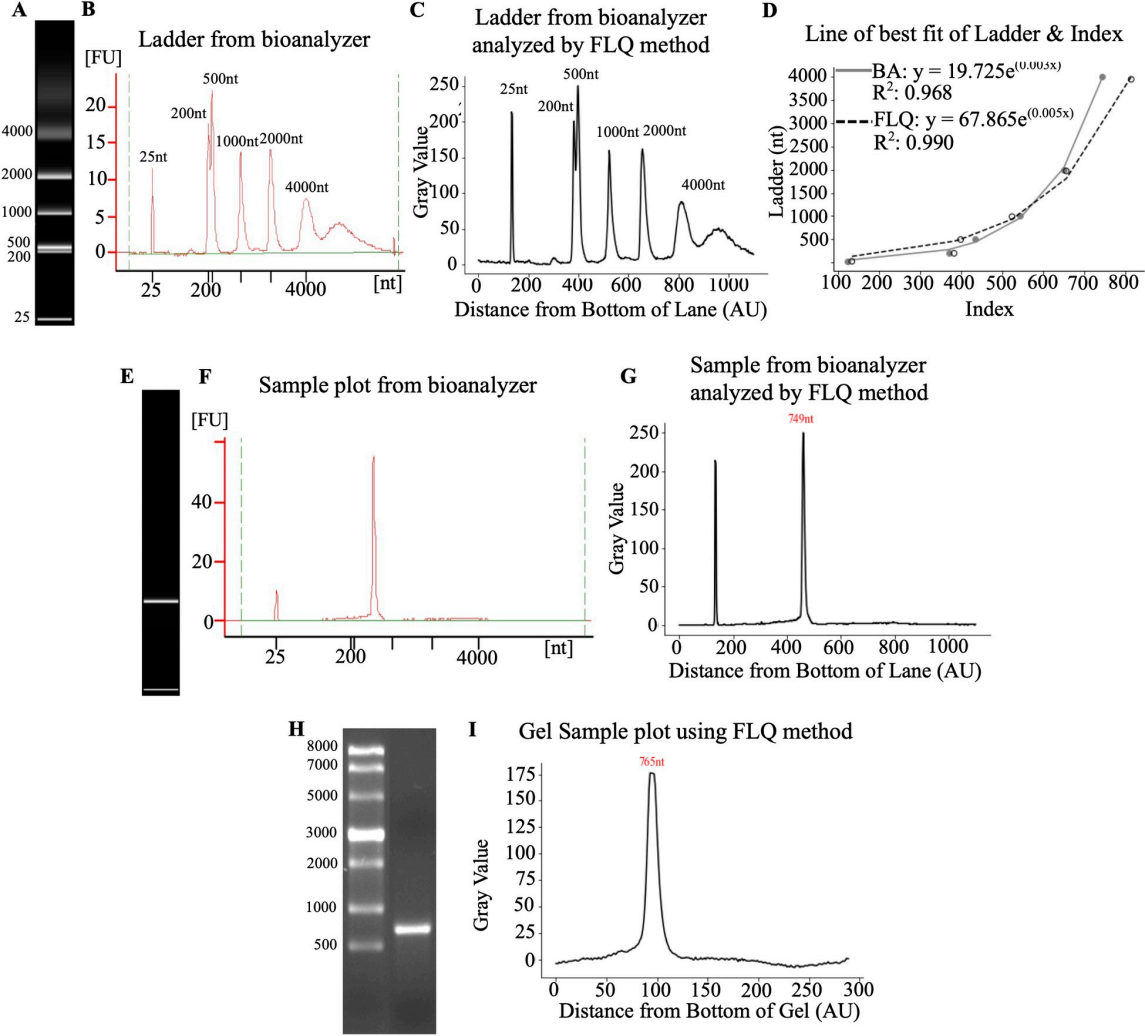
Comparison of FLQ to bioanalyzer. **A)** The image of the ladder obtained from the bioanalyzer. **B)** Ladder profile obtained from bioanalyzer **C)** Ladder profile of bioanalyzer image obtained using FLQ. **D)** Line of the best fit using both methods with the bioanalyzer (BA) data in gray and the FLQ as the dotted line. **E)** mRNA sample image from bioanalyzer chip. **F)** Quantification profile using a bioanalyzer. **G)** Quantification profile of bioanalyzer image using FLQ. **H)** Gel electrophoresis image of the sample. **I)** Quantification of gel electrophoresis image using FLQ. Bioanalyzer data was obtained using 2100 Expert (B.02.08.SI648).

Next, we compared quantifications using the described above FLQ and bioanalyzer. First, we compared the quantification of the image of the molecular weight ladder that was obtained using the Bioanalyzer (Fig 6A). The resulting profiles were identical, which was evident from the similar curves that define molecular weight (Fig 6B and 6C). The Bioanalyzer and FLQ yielded curves where y = 19.725e^(0.003x)^ with an R^2^ = 0.968, and y = 67.865e^(0.005x)^ with an R^2^ = 0.990 respectively (Fig 6D). Similarly, the quantification of the sample from the bioanalyzer (Fig 6E) was the same when using bioanalyzer software (Fig 6F) and FLQ (Fig 6G). Comparing the same sample that was resolved by gel electrophoresis (Fig 6H) quantified using FLQ (Fig 6I) we could see a similar quantification pattern, but the peaks were broader. Suggesting that the resolution of the bioanalyzer pictures is superior to the gel electrophoresis.

### FLQ to RIN direct comparison

**Fig 7:**
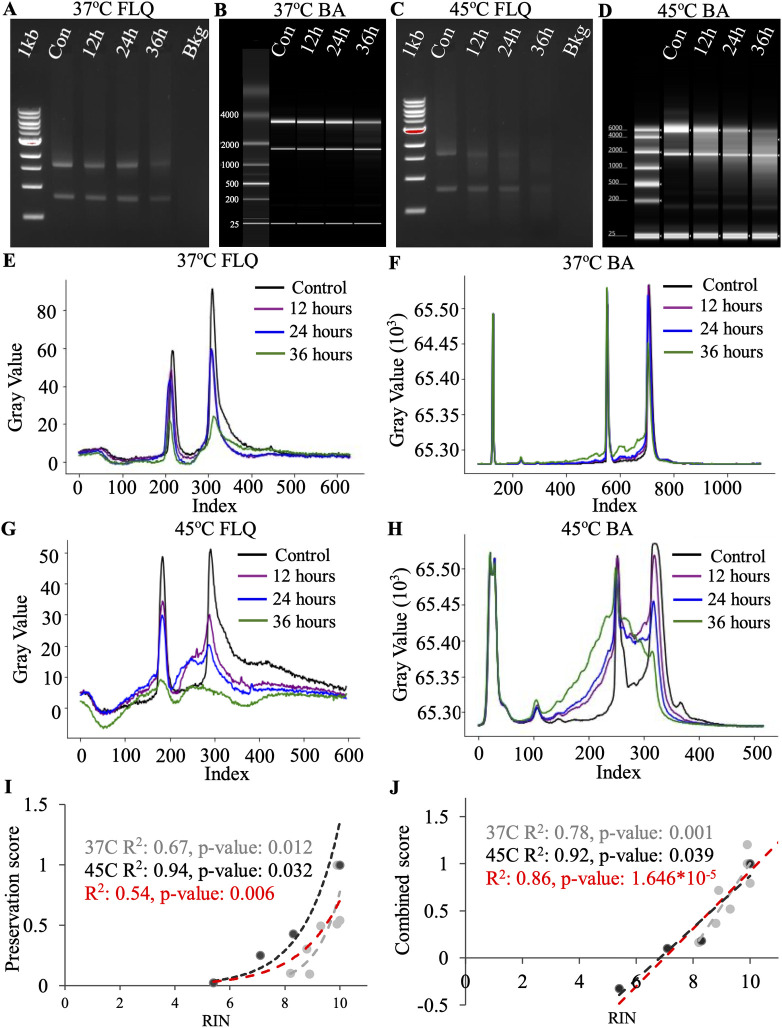
Bioanalyzer RIN comparison to FLQs RIS and preservation score of degraded total RNA. **A)** Representative image of an agarose gel with total RNA after degradation at 37°C. **B)** Bioanalyzer image of total RNA after degradation at 37°C. **C)** Representative image of an agarose gel with total RNA after degradation at 45°C. **D)** Bioanalyzer image of total RNA after degradation at 45°C. **E)** Plot profile of degraded total RNA (37°C) after gel electrophoresis. **F)** Plot profile of degraded total RNA (37°C) after quantification of Bioanalyzer. **G)** Plot profile of degraded total RNA (45°C) after gel electrophoresis. **H)** Plot profile of degraded total RNA (45°C) after quantification of Bioanalyzer. **I)** Scatter plot of the preservation scores of the total RNA. Gray is 37°C incubation, black is 45°C incubation and red includes both temperatures. **J)** Scatter plot of the combined score accounting for the preservation score from the subunit peaks as well as the degradation zones in the areas directly after the peaks. Gray is 37°C incubation, black is 45°C incubation and red includes both temperatures. n = 3.

To correlate the RIN obtained from the bioanalyzer to the FLQ preservation score, we quantified the total RNA after incubation at 37°C and 45°C. Incubation at 37°C led to a mild degradation pattern, as evident from the representative pictures of Agarose gel and bioanalyzer (Fig 7A and 7B). The higher temperature resulted in a pronounced degradation pattern (Fig 7C and 7D). Visually, it appears that no matter the temperature intensity of both 18S and 28S decreases with incubation. The signal quantified by ImageJ demonstrates these decreases during 37°C incubation (Fig 7E). Bioanalyzer quantification showed a similar degradation pattern at the early incubation times at 37°C (Fig 7F). Similarly, signal quantification using ImageJ accurately reproduced the visual representation of the gel picture for 45°C (Fig 7G). Bioanalyzer quantification seemed to be more focused on the degradation part of the signal compared to 18S and 28S peaks (Fig 7H). The FLQ peak preservation number was computed similarly to the method described for IVT. Since total RNA includes two peaks, the AUC for each peak was normalized to the corresponding control. The normalized AUC values of both 18S and 28S peaks were multiplied to obtain a single preservation score per time point. RIN and peak preservation are significantly correlated with each other at 37°C (R^2^: 0.67, p-value: 0.012), 45°C (R^2^: 0.94, p-value: 0.032) and when both temperatures together (R^2^: 0.54, p-value: 0.006) (Fig 7I). The combined FLQ score that utilizes both quantification of the peaks and degradation zones is also significantly correlated with the RIN number (Fig 7J). At 37°C (R^2^: 0.78, p-value: 0.001), 45°C (R^2^: 0.92, p-value: 0.039) and when both temperatures together (R^2^: 0.86, p-value: 1.646*10^−5^).

The exact codes used for the calculations described in the results are appended in S1 Text for Python users and S2 Text for R users.

## Discussion

In this paper, we demonstrate a simple method of quantifying the degradation of *in vitro* transcribed mRNA using gel electrophoresis coupled with ImageJ analysis. We defined it as FLQ because this method capitalizes on the quantification of a signal using the entire lane. This approach allows the selection of various parts of a signal consistently across all lanes within the same gel, thus enabling comparisons of different experimental conditions. Analysis of the data of mRNA degradation for 10 days demonstrated that outcome measures, including AUC of the peak, degradation zone, and combined score, provide sensitive means to evaluate the degradation of mRNA. Typical band quantification protocol requires a user to define the product location within the lane in a gel using reference markers [30]. This task is relatively simple when product size is known. With mRNA degradation, the definition of the product is less trivial.

While the FQL method described in this paper cannot determine the exact quantities of all the different fragments within the sample or the location of the cleavage site, it can provide measures of the degradation profile within each lane. Based on the intensity of the signal, we define the peak of the signal as the position of the most concentrated mRNA of the same size. The width of the signal may vary with application. We used the interval of 20 indexes (+/- 10 around the maximum values). These values can be adjusted based on the resolution of the gel and intensity of the signal. The area under the resulting curve, therefore, represents the quantity of the product within the band. With degradation, the maximal values decrease, leading to reduced corresponding AUC. Of note, if degradation of the product is targeted to the same region of mRNA, it will result in a uniform reduction of the mRNA size that will produce a second band of lower molecular weight and consistent peak shift. Degradation products are seldom the result of very specific cleavage of mRNA and, therefore, the resulting peak is broader than the original product. The degradation of mRNA is underlined by a decrease in the amount of product in each molecular range and the appearance of smaller molecular weight species. Therefore, quantification of both these characteristics encompasses the full range of degradation of mRNA. In our study, we introduced it as a degradation score. Since it incorporates both metrics of the same sample, it results in reduced variability and improved sensitivity of the measurements. Indeed, using the degradation score, we demonstrated significant degradation at each time point. In some applications, mRNA synthesis yields high molecular weight doublets or multiple bands [31]. FLQ method can be used to quantify the appearance of several bands within the same lane. It can be achieved by measuring the AUC of higher molecular weight products.

Often, to improve inter-replicate comparisons, researchers use a normalization procedure expressing the experimental measurements as a fold change relative to the control [32]. We used normalized values of each metric, including peak shift, AUC of the peak, AUC of degradation, and degradation score. We noted that normalization didn’t change the conclusion of statistical analysis about the degree of degradation but reduced the variability between the replicates.

The peak area and preservation scores help to monitor the decrease of that peak position. The smear area/degradation zone and AUC of degradation have high variability due to the larger area and the difference in location where the molecules are cleaved. The variable areas within the degradation zone make it so the normalization to the control may be required to be able to quantify the degradation. The peak shift demonstrates that degradation of the main product occurred and the maximum amount of product shifts from the product peak to the degraded pieces.

It has been demonstrated that when the gel preparation is done carefully, ensuring the agarose is fully dissolved, and the EtBr is dispersed homogeneously, gel electrophoresis can serve as an accurate method for nucleic acid analysis [16]. Agilent 2100 Bioanalyzers can determine concentration measurements within 20% of the UV spectroscopy result and are within 15% accuracy for size measurement compared to the theoretical size [28]. Bioanalyzers use an internal marker and the RNA 6000 Nano ladder in separate channels from the samples to normalize migration times and have a standard for size and quantitation comparison [28]. Bioanalyzers’ data determine RNA quality by using a ratio of the bands from 28S:18S ribosomal RNA but have shown a poor correlation with RNA integrity [17]. For quantification of mRNA out of *in vitro* translation, when only mRNA of interest is present, no RIN number can be acquired. Therefore, the benefit of bioanalyzer takes advantage of a better resolution of the capillary system compared to conventional gel electrophoresis. As we demonstrated in this paper FLQ method yields similar results and may serve as a routine method for mRNA degradation assessment.

Furthermore, the FLQ method can be applied to the quantification of total RNA. In this case, both 18s and 28s peaks and corresponding degradation zones need to be evaluated. The strength of the Agilent bioanalyzer is that it can give an absolute integrity number to any total RNA sample at any given time. FLQ method is based on the comparison to the control condition, which requires samples to be on the same gel. The power of this approach is that it becomes a lot more sensitive and can reveal even very small degradation changes. Using an index based on the reduction of AUC of both 18S and 28S and increases in AUCs of corresponding degradation zones, FLQ can detect the differences in total RNA degradation relative to control, while the bioanalyzer yields the RIN number of each sample independently. Consequently, mild degradation observed in this study was more evident using the FLQ method.

To observe mRNA integrity and quality, other methods, such as denaturing gel electrophoresis, can be used [28, 29]. This method is time-consuming, uses more sample volume than the standard method and utilizes toxic reagents [29]. The reagents that disrupt the secondary structure of mRNA and destroy contaminating RNases impede standard agarose gel electrophoresis [29, 33]. Notably, denaturing can be done by adding bleach to the TAE-agarose mixture to avoid the dangerous and expensive reagents [29]. Denaturing agarose gels and UV spectroscopy will require a large amount of sample, thus a typical electrophoresis method with this evaluation methods may be preferred [28].

To further investigate the capabilities of this quantification method, different concentrations of nucleic acids at various degradation time points could be evaluated to determine concentration as a function of band intensity. Standard gel with EtBr has been successfully used for semi-quantitation of PCR amplicons in a periodontal disease model and image analysis was done with ImageJ [18]. In addition, this method would be extremely useful in techniques such as Electrophoretic Mobility Shift Assays (EMSA) which measure nucleic acid interaction and binding parameters [34]. Utilizing FLQ and acquiring the profile of the entire lane facilitates an efficient evaluation of the migration of a sample. By determining peak shift the mobility shift can easily be quantified. A comparison of peak heights will also indicate the concentrations in each placement from a known initial concentration. Due to the variety of parameters that can be used in EMSA, many buffers, gels, and different methods for visualization, including fluorescent tags and staining, will define the scope of FLQ.

In conclusion, the method described in this paper gives an example of a simple method for signal quantification in the entire lane. We provide well-annotated codes written in R and Python. We used these languages as they gained popularity, and most universities offer programming introductory courses at undergraduate and graduate levels. Any parts of the code can be easily modified to custom fit the needs of a particular experimental setup. ImageJ involves many manipulations and functions, some inherent to the software and others incorporated from user-developed macros and plugins [24, 35, 36]. Plugins are meant to enhance and expand on the capabilities of the software, the most common plugin categories are visualization, preprocessing, segmentation, registration, and tracking [36]. Moreover, in the future, these codes can be linked to ImageJ through an ImageJ Jython Bridge as a plugin to allow users to conduct this analysis within the application [35]. The field lacks a convenient method for evaluating degradation profiles from gel electrophoresis, thus, we developed a comparable method to RINs that is more accessible in addition to providing algorithms to make the analysis more efficient.

## Supporting information

S1 Dataset Fig 1B practice data set. An Excel file output from performing the FLQ method in Fig 1B. This is provided as a sample data set acquired using the FLQ method and is used for the codes provided.
(XLSX)

S2 Dataset All gel data. An Excel file including all the data used to demonstrate the FLQ method, composed of the gel images and the corresponding obtained ImageJ data.
(XLSX)

S1 Text Python code. Annotated Python code. The original code was made in Spyder and annotated in Jupyter notebook. The hash-tagged lines explain the function of the line of code above.
(PDF)

S2 Text R code. Annotated R code. The code was made in R Studio and annotated in R Studio Markdown. The hash-tagged lines explain the function of the line of code above.
(PDF)

S1 File Methods & materials. (PDF)

S1 Raw image Raw images from S2 Dataset. (PDF)
