# Supplementary material for: A convenient analytic method for gel quantification using ImageJ paired with Python or R
Source: PLoS One. 2024 Nov 21;19(11):e0308297. doi: 10.1371/journal.pone.0308297 (PMC11581290; doi:10.1371/journal.pone.0308297)
Supplement: S1 Text — Annotated Python code. The original code was made in Spyder and annotated in Jupyter notebook. The hash-tagged lines explain the function of the line of code above. (PDF) [file pone.0308297.s003.pdf]

Python Gel Quantification Code

Importing packages

In [1]:

```
import pandas as pd
#The pandas package allows user to manage and manipulate datasets.

import numpy as np
#The numpy package allows storage, computation/mathematical functions and
#manipulation of data of the same type within different dimensions (lists,
#tables, etc).

import matplotlib.pyplot as plt
#matplotlib is a library of plotting options for data visualization.
#pyplot is specific collection for charts, graphs and plots. It is

from scipy.signal import find_peaks
#This pckage is used to identify multiple maximum values within a data set
#based on set criteria.

from scipy.optimize import curve_fit
#This package set a line of best fit for a set of data points

from sklearn.metrics import r2_score
#This assess the efficiency of the lines of best fit.

from sklearn.metrics import auc
#The sklearn package accesses a library for data analysis and modeling
#tools used with other packages such as pandas and numpy. Metrics can be
#used for many purposes to evaluate aspects of a models performance, in
#this case to determine the area under curves.

import statistics
#A package loaded for access to basic statistical functions.

import warnings
warnings.filterwarnings('ignore')
#This code is here due to unnecessary warning messages, to view the
#suggestions from the warning simply remove this line of code.
```

Accessing Excel file

In [3]:
```
excel_file = "/Users/Downloads/Supplemental_Figure1b_practicedata_S1.xlsx"
#Replace contents between " " with your file pathway.
#In the top right panel of Console, in the File pane, ensure the desired
#Excel file is accessible within Python. Right click on the file and
#select "Copy Absolute Path".

df = pd.read_excel(excel_file)
#Defines the data frame within Python that copies the excel file.

preview = df.head(3)
#To ensure the file is imported correctly use the head function on the
#dataframe. Index the first few rows (this example 3 rows are evaluated).
```

Option 1: Background Subtraction via Lane Subtraction

In [21]:
```
df1 = df.iloc[0:, 0:]
#Sets up a dataframe for background subtraction by taking every row and
#every column. The [0:] in both cases selects from the first column/row
#to the last that possess data.This can be adjusted to remove data points
#at the tops and/or bottoms of the lanes in the case that the selected
#lane was too long.

df1.columns = ["Distance", "Background", "C1", "E2", "E3", "E4", "ladder"]
#Define the columns within the new data frame df1. In the excel sheet
#there should be a blank lane or an averaged lane from multiple interlane
#spaces, a control, the samples and a ladder.
#REPLACE SAMPLE NAMES WITH OWN DATA.

columns_to_subtract = ["C1", "E2", "E3", "E4", "ladder"]
#Defining columns that the background lane will be subtracted from.
# REPLACE NAMES WITH OWN DATA.
df_b = df1[columns_to_subtract].sub(df1["Background"], axis=0)
#Iterating through each column and using sub function from pandas to
#subtract the background from each row the new data and compiling into
#a new dataframe defined as df_b. The axis = 0 indicates the function
#going along rows/index.
```

Option 2: Background Subtraction from Computation (do not run Option 1)

Calculate gradient in the gel using control for computed background.

Do NOT use Option 2 "Computational Background Subtraction cell" at the same time

In [17]:
```
df_b = df.iloc[0:, 2:]
#Sets up dataframe for background subtraction using computation. This code
#selects all rows and the applicable columns (this could also be [0:, 0:]
#if the excel sheet only include the columns defined below).

df_b.columns = ["C1", "E2", "E3", "E4", "ladder"]
#Define the column names. THESE NAMES & THE COLUMNS NEEDED NEED TO BE
#CHANGED TO MATCH DATA.

control = df_b["C1"]
#Define the control lane from the dataframe using the column name.
#REPLACE TO MATCH YOUR DATA

l = statistics.mean(control[-20:])
#Select the last 20 indices of the lane prior to any bands and take the
#average of the data points.
#THE VALUE WITHIN THE BRACKETS CAN BE CHANGED BASED ON OWN DATA.

f = statistics.mean(control[:50])
#Select the first 50 of the lane after any bands and take the average.
#THE VALUE WITHIN THE BRACKETS CAN BE CHANGED BASED ON OWN DATA.

gr = l/f
#Divide these averages to get a ratio of the beginning and end values of
#the control gel.

df_b['gradient'] = np.linspace(gr, 1, len((df_b)))
#This code adds a new column in the df_b dataframe that includes the
#gradient. The linspace function creates a linear grandient between
#the gr ratio and 1 over the number of indices.

for i in range(0,6):
    df_b.iloc[:,i] = df_b.iloc[:, i]*df_b['gradient']-f
#The first line defines the desired columns to loop the subtraction through,
#and are represented by i. The gradient is multiplied at each point in the
#dataframe then the averaged value of the first 50 rows of the control lane.
#ENSURE THE COLUMNS WITHIN THE (,) MATCH OWN DATA.
```

Defining number of rows/indices

In [5]:
```
df_b['size'] = df_b.index.values
#Adds new column that numbers each row to provide the index.

x_ind = df_b.index.values
#Defines index as an array/list.
```

Plotting background lane with a line of best fit

In [18]:
```
slope, intercept = np.polyfit(x_ind, df1['Background'], 1)
#Using numpy, the polyfit function fits a polynomial of 1 to match the raw
#gel background. This equation uses only slope and y intercept.

line_of_best_fit = slope * x_ind + intercept
#This gets the line equation using the indices as the x-axis.

background = plt.plot(x_ind, df1['Background'], 'k-',
                      label ='Background Gradient')
#Using plt to make a plot of the raw background.

plt.plot(x_ind, line_of_best_fit, color='red', label="Line of best fit")
#Using plt the line of best fit is plotted.

equation_text = f'y = {slope:.2f}x + {intercept:.2f}'
plt.text(400, 31.4, equation_text, fontsize=10, color='red')
#These 2 lines of text define and input the equation of the line on to
#the graph. The (275, 35.4 determines the postion).

plt.xlabel('Distance (AU)')
plt.ylabel('Gray Value')
#Inputs the labels for the x and y axes.

plt.gca().spines['top'].set_visible(False)
plt.gca().spines['right'].set_visible(False)
#Removes the lines on the top and left of the graph.

plt.legend()
#Insert the legend to plot the labels on the graph.
```

Out[18]:
```
<matplotlib.legend.Legend at 0x13c93f8d0>
```

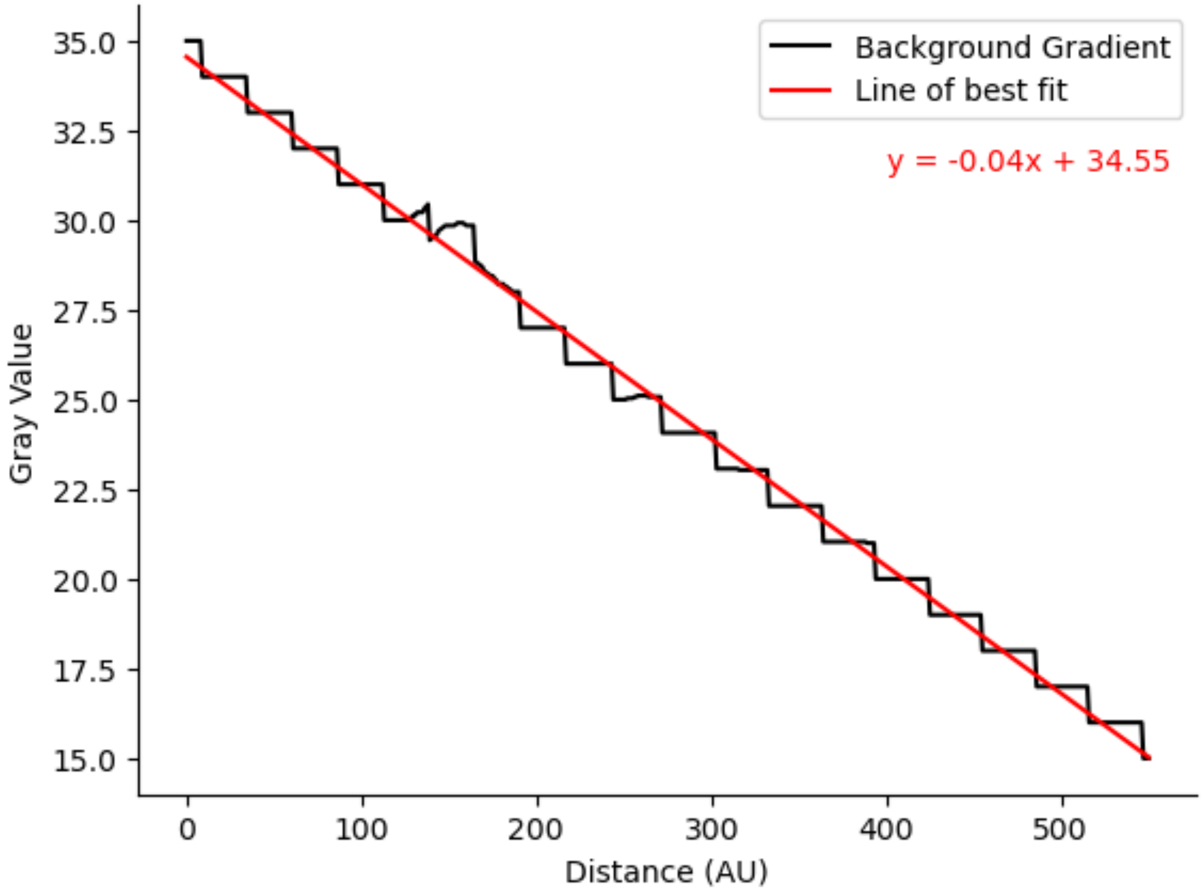

List of maximum index values

In [7]:
```
ind_m_list = []
#Creates an empty list in which to save values.

for i in range(0, 6):
#Loops through columns from df_b. Insert number of columns + 1 (0, x).
    p = np.array(df_b.iloc[:, i])
    #Extracts the data from individual columns.
    max_index = p.argmax()
    #Determines the index with the maximum value in columns.
    ind_m_list.append(max_index)
    #Inserts the maximum values into the empty list.
```

Assigning molecular weights to indices

In [22]:
```
peaks = find_peaks(df_b['ladder'], height= 50, distance= 15)
#This finds the position of peaks within the ladder. Change height and
#peak distance until the correct number of peaks appear in the BasePairs
#dataframe.

peak_indices = peaks[0]
#Lists all the indices where there are peaks in the ladder plot.

ladder_sizes = np.array([500, 1000, 2000, 3000, 5000, 7000])
```

```
#Known ladder sizes. CHANGE DEPENDING ON THE LADDER USED FOR EXPERIMENTS.

BasePairs = {'Column1': peak_indices, 'Column2': ladder_sizes}
bp = pd.DataFrame(BasePairs)
#Makes a data frame of the ladder peaks indices and ladder sizes.


def exponential_model(peak_indices, a, b):
    return a * np.exp(b * peak_indices)
#Defining exponential relationship using the indices of the ladder peaks.

initial_guesses = [1.0, 0.1]
#A list of estimates for a and b parameters.

bounds = ([0, 0], [np.inf, np.inf])
#Contains upper and lower bounds for each parameter.

params, covarince = curve_fit(exponential_model, peak_indices,
                              ladder_sizes,p0=initial_guesses,
                              bounds=bounds)
a, b = params
#This attempts to fit the exponential model to the data with the defined
#parameters.

fitted_y = exponential_model(peak_indices, a, b)
#Defing a variable with the fitted variables using the exponential model.

r_squared = r2_score(ladder_sizes, fitted_y)
print(f'R-squared value: {r_squared}')
#Provides the R^2 value to determine the variance from the line of best fit.

plt.scatter(peak_indices, ladder_sizes, color='black')
#Makes a scatter plot of the values from the laddder peak

plt.plot(peak_indices, fitted_y,
         label=f'Exponential Fit: $y = {a:.3f}e^({b:.3f}x)$', color= 'red')
#Plots fitted line for peak indices.

plt.text(60, 6400, f'R-squared value: {r_squared}', ha='left', va='top',
         color='red', fontsize=12)
#Inserts R^2 value onto the graph.

plt.xlabel('Index')
plt.ylabel('Ladder (bp)')
#Inserts axis labels.

plt.gca().spines['top'].set_visible(False)
plt.gca().spines['right'].set_visible(False)
plt.legend()


ind_bp = []
#Makes an empty list place holder

for item in x_ind:
    y = a * np.exp(b * item)
    ind_bp.append(y)
```

```
#For each value in the variable x_ind the exponential model is applied to
#estimate the amount of nucleotides that corresponds to each index. This
#then fills the empty list.

max_ind_bp = []
#Empty list

for item in ind_m_list:
    y = a * np.exp(b * item)
    max_ind_bp.append(y)
#Loops through maximum peak indices with expontnial model intrapolate
#the number of nucleotides.
```

```
R-squared value: 0.9901697138627319
```

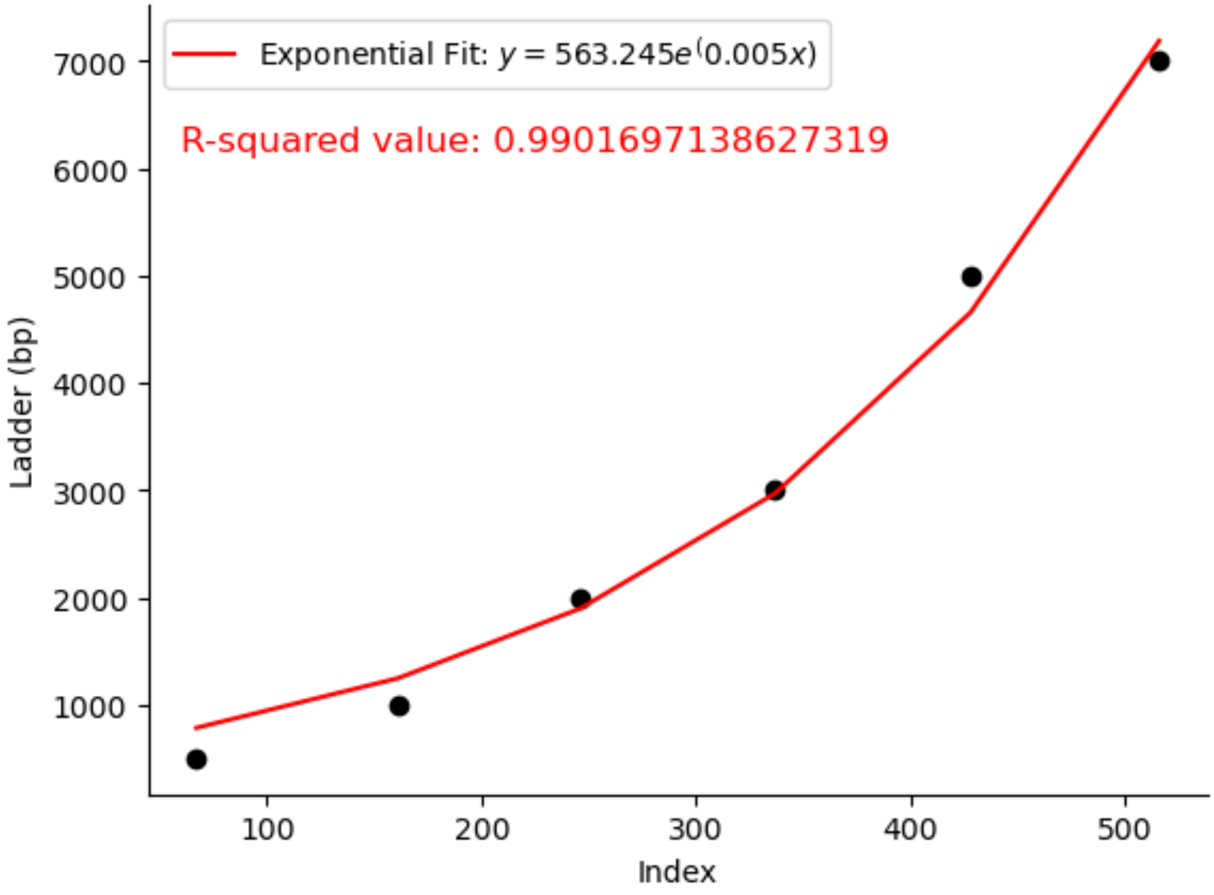

Making plots

In [23]:
```
ladder = plt.plot(x_ind, df_b['ladder'], 'k-', label = 'Ladder')
#Plots Gray value as a function of the size of the gel/by BP where 'k-'
#makes the line black and solid. Repeat this line of code for all
#groups to plot them. ENSURE COLUMN NAMES MATCH DATAFRAME.

plt.xlabel('Index')
plt.ylabel('Gray Value')
plt.legend()
#If all groups are plotted together the label of each plot will also be
#plotted on the graph.
plt.gca().spines['top'].set_visible(False)
plt.gca().spines['right'].set_visible(False)
```

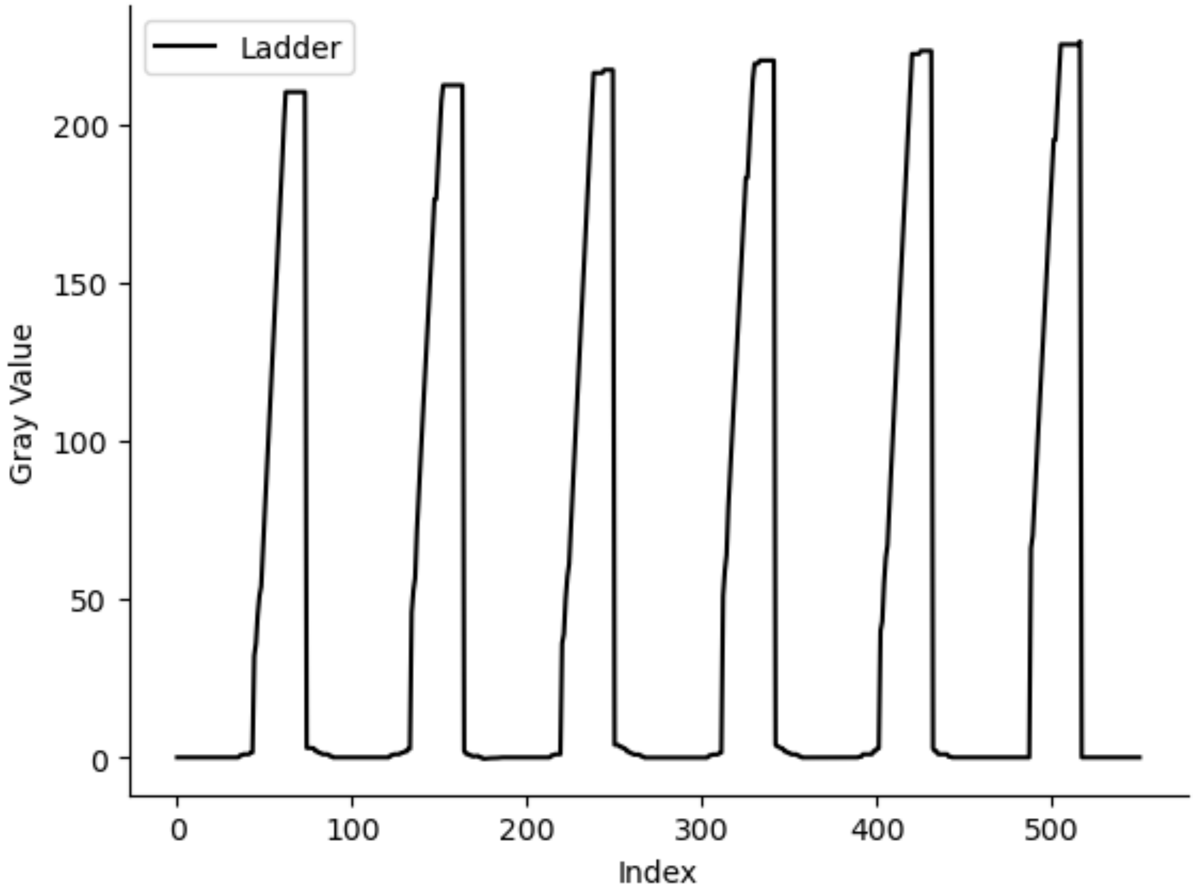

List of means

In [10]:
```
mean_list = np.mean(df_b.iloc[:, 0:6], axis=0).tolist()
#This code uses numpy to take the mean of each column in the dataframe df_b
#including all rows. Axis = 0 dictatesthat the means are calculated along
#the columns. This is all added into a list.
```

List of areas under the curve

In [11]:
```
auc_list = []
#Empty list

for i in range(0,6):
    #Loops through columns of df_b.
    c = auc(x_ind, df_b.iloc[:, i])
    #Calculates the area under of the curve based on the indices and Gray
    #values (x,y).
    auc_list.append(c)
    #Adds values to empty list.
```

Area under product peaks only

In [12]:
```
x_p = ind_m_list[0]
#Maximum value of the first column in df_b (the control group).

band_l = x_p -10
#Selects specific x-axis values around peak in the negative direction, this
#number is chosen to fit the control peak.

band_r = x_p +10
#Same as line above but in the opposite direction.

df_p = df_b.iloc[band_l:band_r, :]
#Selects the rows around the maximum value defined by the 2 lines above to
#create a dataframe that includes only the defined values for all columns.

df_p['size']=df_p.index.values
#Defines the size of peaks as an index and adds to the data frame as a
#column.

mean_p = np.mean(df_p.iloc[:, 0:6], axis=0).tolist()
#This code calculates the mean of each column of df_p (the dataframe of
#only the peak region and adds to a new list.

auc_p_list = []
#Empty list
for i in range(0,6):
#Loops through columns of df_p.
    a_p = auc(df_p['size'], df_p.iloc[:, i])
    #Calculates the area under of the curve of the peak.
    auc_p_list.append(a_p)
    #Adds peak areas to the list.
```

Area under the smear/degradation zone (region before product peak)

In [13]:

```
smear_condition = np.logical_and(0 <= x_ind, x_ind <= band_l)
#Defines a condition where the x-axis is equal to or larger than 0 but
#less than the start value of the product peak.

df_s = df_b[smear_condition]
#Applys the condition to make a new dataframe for the smear.

df_s['size']=df_s.index.values
#Makes new column with the indices of the smear.

mean_s = np.mean(df_s.iloc[:, 0:6], axis=0).tolist()
#This code calculates the mean of each column of df_s (the dataframe of
#only the peak region and adds to a new list.

auc_s_list = []
#Empty list
for i in range(0,6):
#Loops through columns of df_s.
    a_s = auc(df_s['size'], df_s.iloc[:, i])
    #Calculates the area under of the curve of the degradation zone.
    auc_s_list.append(a_s)
    #Adds values to the list.
```

Normalizing values to the control

In [14]:

```
n_mean = list(map(lambda m: m/mean_list[0], mean_list))
#The lambda function divides m value by the first value in the list
#(aka the control [0]). Using (map()) the function is applied to
#each value in the list. All values are sorted in a new list.

n_auc = list(map(lambda u: u/auc_list[0], auc_list))
#Same as line above but for the areas under the curve.

n_mean_p = list(map(lambda n: n/mean_p[0], mean_p))
#Same as lines above but for the peak AUC.

n_mean_s = list(map(lambda n: n/mean_s[0], mean_s))
#Same as lines above but for the degradation zone AUC.

preservation = [num/auc_p_list[0] for num in auc_p_list]
#Divides each product peak value in the list by the first
#(the control [0]).

degradation = list(map(lambda a_s: a_s/auc_s_list[0], auc_s_list))
#Diviedes each degradation zone value in the list by the control.

peak_shift = [num/ind_m_list[0] for num in ind_m_list]
#Same as line above but for the index directly to get a ratio that
#demonstrates the relative position of all samples maximum peak
#compared to the control.
```

Define degradation

In [15]:
```
deg = [a*b for a, b in zip(n_mean, n_auc)]
#Creates a new list of the products of the mean and area under the curve at
#each position.

deg_p = [a*b for a, b in zip (n_mean_p, preservation)]
#Same as above but only for peak area.
```

Output into excel sheet

In [16]:
```
groups = ["C1", "E2", "E3", "E4"]
#CHANGE TO MATCH YOUR DATA.
timepoints = [0, 2, 4, 7, 10]
#In the case of multiple conditions the timepoints must be repeated for
#each condition ([0, 2, 4, 7, 10, 0, 2, 4, 7, 10, ...], this example does
#not require specified timepoints and only uses one condition.

df_a = pd.DataFrame(list(zip(groups, max_ind_bp, mean_list, auc_list,
                             mean_p, auc_p_list, deg, deg_p, peak_shift,
                             timepoints)), columns = ["groups", "BP",
                                                      "mean", "auc",
                                                      "mean_p", "auc_p",
                                                      "deg", "deg_p",
                                                      "peak_shift", "Time" ]
#Combines all the lists previously defined into a new dataframe.

df_a.reset_index(drop = True, inplace = True)
#Makes a new index for this dataframe.

df_a.to_excel('Gel#_quantified_date.xlsx', index=False)
#Converts the dataframe to an excel sheet. Replace contents between ' '
#with the desired document name.
```
