# Supplementary material for: A convenient analytic method for gel quantification using ImageJ paired with Python or R
Source: PLoS One. 2024 Nov 21;19(11):e0308297. doi: 10.1371/journal.pone.0308297 (PMC11581290; doi:10.1371/journal.pone.0308297)
Supplement: S2 Text — Annotated R code. The code was made in R Studio and annotated in R Studio Markdown. The hash-tagged lines explain the function of the line of code above. (PDF) [file pone.0308297.s004.pdf]

R Gel Quantification Code

```
LOADING PACKAGES

install.packages("readxl")
library(readxl)
# These 2 lines install and load the package to access excel files (.xls and .xlsx
formats).

install.packages("openxlsx")
library(openxlsx)
# These 2 lines install and load the package to access excel files (.xlsx and .xlsm
formats).

install.packages("dplyr")
library(dplyr)
# These lines install and load a package used for data management and analysis using
basic functions to manipulate the data.

library(data.table)
# This package is used to optimize manipulation of large data sets, key functions
being indexing and joining.

library(ggplot2)
# This package is used for data visualization in the form of various plot types,
particularly scatter and bar graphs for this case.

install.packages("pROC")
library(pROC)
# This package is typically used for analyzing and visualizing receiver operating cha
racteristics curves but it also includes a function to calculate the area under the
curve (AUC) which was of interest in this case.

install.packages("pracma")
library(pracma)
# This package provides a variety of mathematical operations, computations not
included in baseline R, and optimization functions.

install.packages("DescTools")
library(DescTools)
# This package is used for statistical analysis and data visualization.
```

```
UPLOADING THE EXCEL FILE

excelfile <- read_xlsx("Users/Downloads/Supplemental_Figure1b_practicedata_S1.xlsx")
# This line calls on the desired excel file. To get the file pathway go to the
'Files' tab in the console below the global environment. Find the desired document,
click so the options 'View File' and 'Import Data set' appear. Select 'Import Data
set', copy the pathway within the code preview and paste in the (" ").

head(excelfile, 3)
# This code previews the first 3 rows of the excel file to confirm the data.
```

```
OPTION 1: BACKGROUND SUBTRACTION VIA LANE SUBTRACTION

df <- excelfile[c(2, 3, 4, 5, 6, 7)]
# This code selects 6 columns of the excel sheet, excluding the first column which
is the Distance of the gel from ImageJ. CHANGE TO MATCH USER'S OWN DATA SET.

df <- df %>%
  slice(0:n())
# These two lines of code select for particular rows within the data frame, this can
be used in the case that the lane selection was too long at the top or bottom of the
lane.

colnames(df) <- c("Background", "C1", "E2", "E3", "E4", "ladder")
# This line defines the columns within the data frame. The background lane is only
applicable if option 1 is the desired method. CHANGE TO MATCH USER'S OWN DATA SET.

columns <- c("C1", "E2", "E3", "E4", "ladder")
df_b <- df[, columns] - df$Background
# Subtracts the background lane from each of the other lanes. CHANGE TO MATCH USER'S
OWN DATA SET.
```

```
OPTION 2: BACKGROUND SUBTRACTION VIA COMPUTED GRADIENT

df <- excelfile[c(3, 4, 5, 6, 7)]
# This code selects 5 columns of the excel sheet, excluding the first two columns
which is the Distance of the gel from ImageJ and the Background lane (if using
this option then this would not be a lane present).CHANGE TO MATCH USER'S OWN DATA
SET.

df <- df %>%
  slice(0:n())
# These two lines of code select for particular rows within the data frame, this can
be used in the case that the lane selection was too long at the top or bottom of the
lane.

colnames(df) <- c("C1", "E2", "E3", "E4", "ladder")
# Redefines the column names to be called upon later. CHANGE TO MATCH USER'S OWN DATA
SET.


l <- mean(tail(df$C1, 50))
# Selects the last 50 indices of the control lane prior to any bands and takes the
average of the data points. This can be defined by the user.

f <- mean(head(df$C1, 50))
# Selects the first 50 of the control lane after any bands and takes the average.
This can be defined by the user.

gr <- mean(l/f)
# Divide these averages to get a ratio of the beginning and end values of the control
gel.

df$gradient <- seq(gr, 1, length.out = nrow(df))
# This line creates a new column called 'gradient' that starts at the gr value and
goes to 1 in the data frame 'df'.

apply_equation <- function(column) {
  return ((column * df$gradient) - f)
}
# The first line defines a function to be applied column-wise manner. The second line
defines the function so that each column is multiplied by the previously defined
gradient and then subtracted by the averaged first 50 points of the control lane.

columns_to_loop <- c("C1", "E2", "E3", "E4", "ladder")
# Defining the columns to subtract the backgrounds from. CHANGE TO MATCH USER'S OWN
DATA SET.

df_b <- as.data.frame(lapply(df[columns_to_loop], apply_equation))
# Creating a new data frame.
```

```
DEFINING THE NUMBER OF ROWS

df_b$size <- rownames(df_b)
# Adds new column that numbers each row to provide the index/defining the number of
rows.

x_ind <- as.numeric(rownames(df_b))
# Defines index as an array/list.
```

```
PLOTTING THE BACKGROUND LANE WITH A LINE OF BEST FIT AFTER OPTION 1

fit <- lm(df$Background ~ x_ind)
# Fits linear regression model using the background lane and Index.

slope <- coef(fit)[2]
intercept <- coef(fit)[1]
# Defines the slope and intercept of the linear regression model.

line_of_best_fit <- slope * x_ind + intercept
# Generate the line of best fit using the model.

cat("Equation of the line of best fit: y =", round(slope, 2), "* x +", round(intercep
t, 2), "\n")
# cat allows values to be printed. This line of codes also rounds the values to two
decimal points.


plot(x_ind, df$Background, type='l', col='black', xlab='Distance (AU)', ylab='Gray Va
lue', main='Background Gradient')
# Plot background, type 'l' defines the points be connected by straight lines.

lines(x_ind, line_of_best_fit, col='red', lty=1, lwd=2)
# Plots the line of best fit. lty makes the line straight and lwd determines the line
width.

legend('topright', legend=c('Background', 'LOB = -0.04 * x + 34.55'), col=c('black',
'red'), lty = 1, bty = "n")
# This defines the legend and its placement.
```

```
FINDING THE MAXIMUM VALUES IN EACH COLUMN

max_values_list <- numeric()
# Creates an empty list in which to save values.

for (col in names(df_b)[1:5]) {
# Loops through all the columns of df_b.
  max_values <- max(df_b[[col]])
# Calculates the index for the maximum value in columns.
  max_values_list <- c(max_values_list, max_values)
# Inserts the maximum values into the empty list.
}
```

```
IDENTIFYING THE INDEX OF THE MAXIMUM VALUES

ind_m_list <- integer()
# Creates an empty list in which to save values.

for (i in 1:5) {
# Loops through all the columns of df_b.
  p <- unlist(df_b[, i])
# Extracts the data from individual columns.
  max_index <- which.max(p)
# Determines the index with the maximum value in columns.
  ind_m_list <- c(ind_m_list, max_index)
# Inserts the maximum values into the empty list.
}
```

```
ASSIGNING INDICES AS MOLECULAR WEIGHTS

peaks <- findpeaks(df_b$ladder, minpeakheight = 50, minpeakdistance = 14)
# This finds the position of peaks within the ladder. Change height and peak distance
until the correct number of peaks appear in the BasePairs data frame.

peak_indices <- sort(peaks[,2])
# Selects the column of the 'peaks' array where the peak position from the index is
stored.

ladder_sizes <- c(500, 1000, 2000, 3000, 5000, 7000)
# Known ladder sizes. CHANGE DEPENDING ON THE LADDER USED FOR EXPERIMENTS.

BasePairs <- data.frame(Column1 = peak_indices, Column2 = ladder_sizes)
# Makes a data frame of the ladder peaks indices and ladder sizes.


exponential_model <- function(x, a, b) {
  return(a * exp(b * x))
}
```

```
# Defines the exponential model.

initial_guesses <- list(a = 1000, b = 0.01)
# A list of estimates for a and b parameters.

fit <- nls(ladder_sizes ~ a * exp(b * peak_indices), start = initial_guesses, lower =
c(0, 0), upper = c(Inf, Inf), algorithm = "port")
# Fits data to the model. Includes defined upper and lower bounds using the "port"
algorithm.

a <- coef(fit)['a']
b <- coef(fit)['b']
# Extracts parameters from the fit.

fitted_values <- predict(fit)
# Calculates fitted values for ladder size to the peak indices.

SSR <- sum((ladder_sizes - fitted_values)^2)
# Calculates the Sum of Squared Residuals.

SST <- sum((ladder_sizes - mean(ladder_sizes))^2)
# Calculates the Total Sum of Squares.

r_squared <- 1 - SSR / SST
# Calculates the R^2 value to determine how well the curve fits.

cat(paste("a =", round(a, 3), "\n"))
cat(paste("b =", round(b, 3), "\n"))
# Prints the a and b parameters rounded to 3 decimal points.

cat(paste("R-squared value:", round(r_squared, 3), "\n"))
# Prints the R^2 value rounded to 3 decimal points.


plot(peak_indices, ladder_sizes, pch = 16, col = "black", xlab = "Peak Indices", ylab
= "Ladder Sizes", main = "Exponential Curve of Best Fit")
# Plot the data.

lines(peak_indices, fitted_values, col = "red", lty = 1, lwd = 2)
# Plot the fitted curve on the data.

legend("topleft", legend = c("Fitted Curve"), col = c("red"), lty = c(1), bty = "n")
# Plot the legend for the Fitted curve. Set bty to "n" to remove the defaulted box
around the legend.

equation <- sprintf("y = %.3f * exp(%.3f * x)", a, b)
# Defines the equation of the curve.

text(250, 5500, equation, col = "black")
# Plots the equation onto the graph.
```

```
text(227, 4500, paste("R^2 =", round(r_squared, 3)), col = "black")
# Plots the R^2 value onto the graph.

ind_bp <- sapply(x_ind, function(item) a * exp(b * item))
# For each value in  x_ind the exponential model is applied to estimate the amount of nucleotides that corresponds to each index.

max_ind_bp <- sapply(ind_m_list, function(item) a * exp(b * item))
# Applies the exponential model to each value stored in ind_m_list which is the index position of each peak from all the lanes in the data frame.
```

```
MAKING PLOTS

plot(x_ind, df_b$C1, type = "l", col = "black", ylim = c(min(df_b$C1), max(df_b$C1)),
     xlab = "Index", ylab = "Grey Value", main = "Control")
# Plots Gray value as a function of the size of the gel/by BP. Repeat this line of code for all groups to plot them. ENSURE COLUMN NAMES MATCH DATAFRAME.
```

```
LIST OF MEANS

means <- apply(df_b[1:5], 2, mean)
# This determines the mean of each column in the dataframe df_b.
```

```
AREAS UNDER THE CURVE (AUC)

auc_values_list <- numeric()
# Empty list

for (col in names(df_b)[1:5]) {
# Loops through columns of df_b.
  auc_value <- AUC(x_ind, df_b[[col]])
# Calculates the area under of the curve based on the index and Gray values from each column (x,y).
  auc_values_list <- c(auc_values_list, auc_value)
# Adds values to empty list.
}
```

```
DEFINING THE REGION OF THE PEAKS

x_p <- ind_m_list[[1]]
# Maximum value of the first column in df_b (the control group).

band_l <- x_p - 10
# Selects specific x-axis values around peak in the negative direction, this
number is chosen to fit the control peak.

band_r <- x_p + 10
# Same as line above but in the opposite direction.

df_p <- df_b[band_l:band_r, ]
# Selects the rows around the maximum value defined by the 2 lines above to create a
data frame that includes only the defined values for all columns.

df_p$size <- rownames(df_p)
# Defines the size of peaks as an index and adds to the data frame as a column
```

```
MEAN OF PEAKS

mean_p <- apply(df_p[1:5], 2, mean)
# This determines the mean of peak region of each column from the data frame df_p.
```

```
AUC OF PEAKS

auc_p_list <- numeric()
# Empty list

xpeak_ind <- as.numeric(rownames(df_p))
# Defines index as an array/list to match the data frame made for the region of the
peaks.

for (i in 1:5) {
# Loops through all the columns of df_p.
  a_p <- AUC(xpeak_ind, df_p[, i])
# Calculates the area under of the curve based on the index and Gray values from each
column (x,y).
  auc_p_list <- c(auc_p_list, a_p)
# Adds values to empty list.
}
```

```
DEFINING THE REGION OF THE SMEAR/DEGRADATION ZONE

smear_condition <- df_b$size >= 0 & df_b$size <= band_l
# Defines a condition where the x-axis is larger than 0 but less than the start value
of the product peak.

df_s <- df_b[smear_condition, ]
# Applys the condition to make a new data frame for the smear.

df_s$size <- rownames(df_s)
# Makes new column with the indices of the smear.

xsmear_ind <- as.numeric(rownames(df_s))
# Defines index as an array/list to match the data frame made for the region of the
smear/degradation zone.
```

```
MEAN OF THE SMEAR/DEGRADATION ZONE

mean_s <- apply(df_s[1:5], 2, mean)
# This determines the mean of degraded region of each column from the data frame df_s
.
```

```
AUC OF THE SMEAR/DEGRADATION ZONE

auc_s_list <- numeric()
for (i in 1:5) {
# Loops through all the columns of df_ps
  a_s <- AUC(xsmear_ind, df_s[, i])
# Calculates the area under of the curve based on the index and Gray values from each
column (x,y).
  auc_s_list <- c(auc_s_list, a_s)
# Adds values to empty list.
}
```

```
NORMALIZING VALUES TO THE CONTROL

n_mean <- means/means[1]
# Divides each mean value in the list by the first value aka the control.

n_auc <- auc_values_list / auc_values_list[1]
# Divides each AUC value in the list by the first value aka the control.

n_mean_p <- mean_p / mean_p[1]
# Divides each peak mean value in the list by the first value aka the control.

n_mean_s <- mean_s / mean_s[1]
# Divides each smear/degradation zone mean value in the list by the first value aka
the controL.


preservation_score <- auc_p_list/ auc_p_list[[1]]
# Divides each peak AUC value in the list by the first value aka the control.

degradation_score <- auc_s_list / auc_s_list[[1]]
# Divides each smear/ degradation zone AUC value in the list by the first value aka
the control.


peak_shift <- ind_m_list / ind_m_list[1]
# Divides each peak index by the control to obtain a ratio that indicates the shift
from the controls position.
```

```


deg <- n_mean * n_auc
# Creates a new list of the products of the mean and area under the curve at each
position.

deg_p <- n_mean_p * preservation_score
# Same as above but only for peak area.
```

```
groups <- c("C1", "E2", "E3", "E4", "ladder")
# Defines the groups which will be shown as a column in exported excel file. CHANGE
TO MATCH USER'S DATA.

timepoints = c(0, 2, 4, 7, 10)
# Time points can also be added as a column by adding this variable into the
dataframe below. In the case of multiple conditions the time points must be
repeated for each condition ([0, 2, 4, 7, 10, 0, 2, 4, 7, 10, ...], this example
does not require specified time points and only uses one condition.

df_a <- data.frame(
  groups = get("groups", envir = .GlobalEnv),
  BP = get("max_ind_bp", envir = .GlobalEnv),
  mean = get("means", envir = .GlobalEnv),
  auc = get("auc_values_list", envir = .GlobalEnv),
  mean_p = get("mean_p", envir = .GlobalEnv),
  auc_p = get("auc_p_list" , envir = .GlobalEnv),
  mean_s = get("mean_s", envir = .GlobalEnv),
  auc_s = get("auc_s_list", envir = .GlobalEnv),
  pres_sc = get("preservation_score", envir = .GlobalEnv),
  deg_sc = get("degradation_score", envir = .GlobalEnv),
  deg = get("deg", envir = .GlobalEnv),
  deg_p = get("deg_p", envir = .GlobalEnv),
  ps = get("peak_shift", envir = .GlobalEnv)
  )
# Combines all the lists previously defined in the Global Environment into a new data
frame.

write.xlsx(df_a, "Gel#_quantified_date.xlsx", sheetName = "Sheet1", rowNames = FALSE)
# Converts the dataframe to an excel sheet. Replace contents between ' ' with the
desired document name.
```
