# Supplementary material for: A convenient analytic method for gel quantification using ImageJ paired with Python or R
Source: PLoS One. 2024 Nov 21;19(11):e0308297. doi: 10.1371/journal.pone.0308297 (PMC11581290; doi:10.1371/journal.pone.0308297)
Supplement: S1 File — (PDF) [file pone.0308297.s005.pdf]

protocols.io Part of SPRINGER NATURE

A Convenient Analytic Method for Gel Quantification Using ImageJ Paired with Python or R.

RESERVED DOI:
10.17504/protocols.io.81wgbz4dygpk/v1

Cassidy Tomlinson[1,2], Ashwini Rajasekaran[1,2], Ariane Khatiz[1], A. James Farmilo[2], Pavel Gris[2], Marjo Piltonen[2], Amanda Matthews[2], Abdelaziz Amrani[1], Denis Gris[1,2]

[1]Université de Sherbrooke; [2]deutraMed

Cassidy Tomlinson
Université de Sherbrooke

Protocol Info: Cassidy Tomlinson, Ashwini Rajasekaran, Ariane Khatiz, A. James Farmilo, Pavel Gris, Marjo Piltonen, Amanda Matthews, Abdelaziz Amrani, Denis Gris . A Convenient Analytic Method for Gel Quantification Using ImageJ Paired with Python or R.. **protocols.io** **https://protocols.io/view/a-convenient-analytic-method-for-gel-quantificatio-dgdd3s26**

Created: June 27, 2024

Last Modified: June 27, 2024

Protocol Integer ID: 102533

Keywords: Gel quantification, ImageJ, Python, R, Analytic method

Funders Acknowledgement:
AA Engineering Research Council of Canada
Grant ID: RGPIN-2015-03671
MITACS Accelerate
Grant ID: RGPIN-2019-05285

Abstract

This protocol details the convenient analytic method for gel quantification using ImageJ paired with Python or R.

Materials

In-Vivo transcription - mixture:

A	B
linearized DNA	1 µg
each nucleotide (A, G, C, U-TP)	2 µL
reaction buffer	2 µL
polymerase	2 µL
RNase-free H2O	8 µL

QIAGEN RNeasy Kit (#74136) and TRIzol® (#15596018) RNeasy Plus Mini Kit (250) **Qiagen Catalog #**74136

TRIzol™ Reagent **Thermo Fisher Catalog #**15596018

Bacterial Transformation

2d 1h 15m

1 Competent E. coli cells in glycerol were thawed from -80 °C .

2 In a falcon tube 20 µL – 50 µL of cells were incubated with 10 ng – 100 ng of DNA encoding for human NLRx1 variant 4 then were transformed via heat shock in a water bath at 42 °C .

3 The transformed cells were plated and incubated at 37 °C for 24:00:00 in LB agar containing ampicillin.

1d

4 3-4 colonies were inoculated into liquid LB media with ampicillin and were left shaking at 37 °C for 24:00:00 .

1d

5 The cells were separated from the LB media with 6000 rpm, 4°C, 00:15:00 .

15m

6 Following the GenElute Maxiprep protocol, the DNA was isolated from the cells. The concentration was read in a BioDrop analyser.

7 The 10 µg of DNA was linearized using 2 µL of restriction enzyme, 10 µL of cutSmart buffer and filled up to 100 µL of H2O. This was incubated at 37 °C for 01:00:00 . The DNA was then purified using the QIA Prepkit and the yield was again read in the BioDrop analyser.

1h

In vitro transcription

2h

8 A mixture of 1 µg of linearized DNA, 2 µL of each nucleotide (A, G, C, U-TP), 2 µL of reaction buffer, 2 µL of polymerase, and 8 µL of RNase-free H2O was, vortexed and incubated for 02:00:00 at 37 °C .

2h

A	B
linearized DNA	1 µg
each nucleotide (A, G, C, U-TP)	2 µL
reaction buffer	2 µL
polymerase	2 µL
RNase-free H2O	8 µL

RNA Extraction

9 Total RNA was extracted from U118mg and U87mg cell lines obtained from ATCC by following the standard protocols for QIAGEN RNeasy Kit (#74136) and TRIzol® (#15596018)

10 The concentrations were measured in a BioDrop spectrophotometer.

Degradation

11 The total RNA samples were incubated at 37 °C or 45 °C for the allotted time points, 0 days, 2 days, 4 days, 7 days, and 10 days or 0 hours, 3 hours, 6 hours, 12 hours, 24 hours, 36 hours, and 48 hours.

12 The IVT mRNA samples were incubated at 37 °C for the allotted time points, 0 days, 2 days, 4 days, 7 days, and 10 days.

Agarose Gel electrophoresis

45m

13 Gel electrophoresis was performed using 2% agarose in a buffer containing 1xTris, acetate, and ethylenediaminetetraacetic acid (EDTA) (TAE) and 5 µL EtBr.

14 The gel electrophoresis was conducted at 96V for ~ 00:45:00 .

45m

15 Digital images were acquired with BioRad GelDoc.

Analysis with Image J:

16 To open an image File>open.

17 Turn the image black and white Image> type>16 bit (dark background and light bands).

18 To acquire the entire lane signal, the gel needs to be rotated 90 degrees; image>transform>rotate 90° right.

19 Select the rectangle tool and draw a box around the first lane (typically the molecular weight ladder) so that the box includes the entire lane length, as shown in Fig 10.

20 To show the graph, click Analyze>Plot profile.

21 To get data click Live>List. Only click Live for the first lane.

22 Copy the whole list into an Excel sheet and close the list.

23 Hover the cursor over the box outline until the pointer appears and click. Use arrow keys to move the box to the next lane, do not use the left or right arrows.

24 Repeat steps 21-23. Maintain the box and measure a blank lane; if all lanes are used, the box can be narrowed to measure 3-5 spaces between lanes (box width will need to be adjusted). Alternatively, the background can be computed using top and the bottom grey values of the gel.

25 Ensure that the Excel sheet has columns in the following order: distance, background (if measuring directly), nucleic acid ladder, control, and each time point. then add any experimental conditions such as solvent, transcript differences, temperature, etc. Include the gel name/number and date in the file name.

Codes

26 The script for Python was developed in the environment Spyder.

27 The script for R was written in RStudio.

28 The corresponding script to the desired programming software must be used.

29 The data file pathway must be changed to match the desired Excel file.

30 The indicated lines of code must be modified to match the user’s own data sets (number of columns/rows, column names, etc).
