# Supplementary material for: A convenient analytic method for gel quantification using ImageJ paired with Python or R
Source: PLoS One. 2024 Nov 21;19(11):e0308297. doi: 10.1371/journal.pone.0308297 (PMC11581290; doi:10.1371/journal.pone.0308297)
Supplement: S1 Raw image — (PDF) [file pone.0308297.s006.pdf]

IVT_37_n1

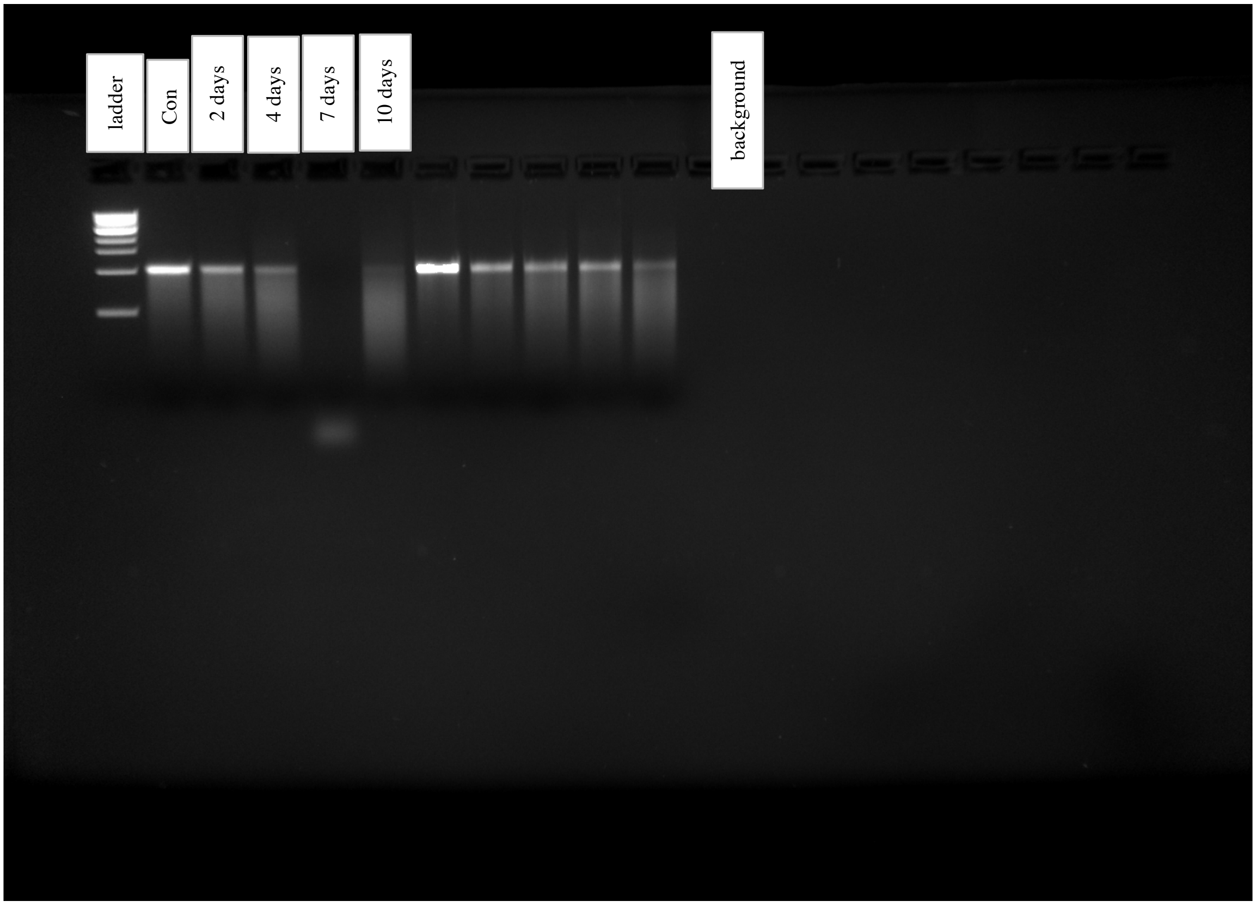

IVT_37_n2

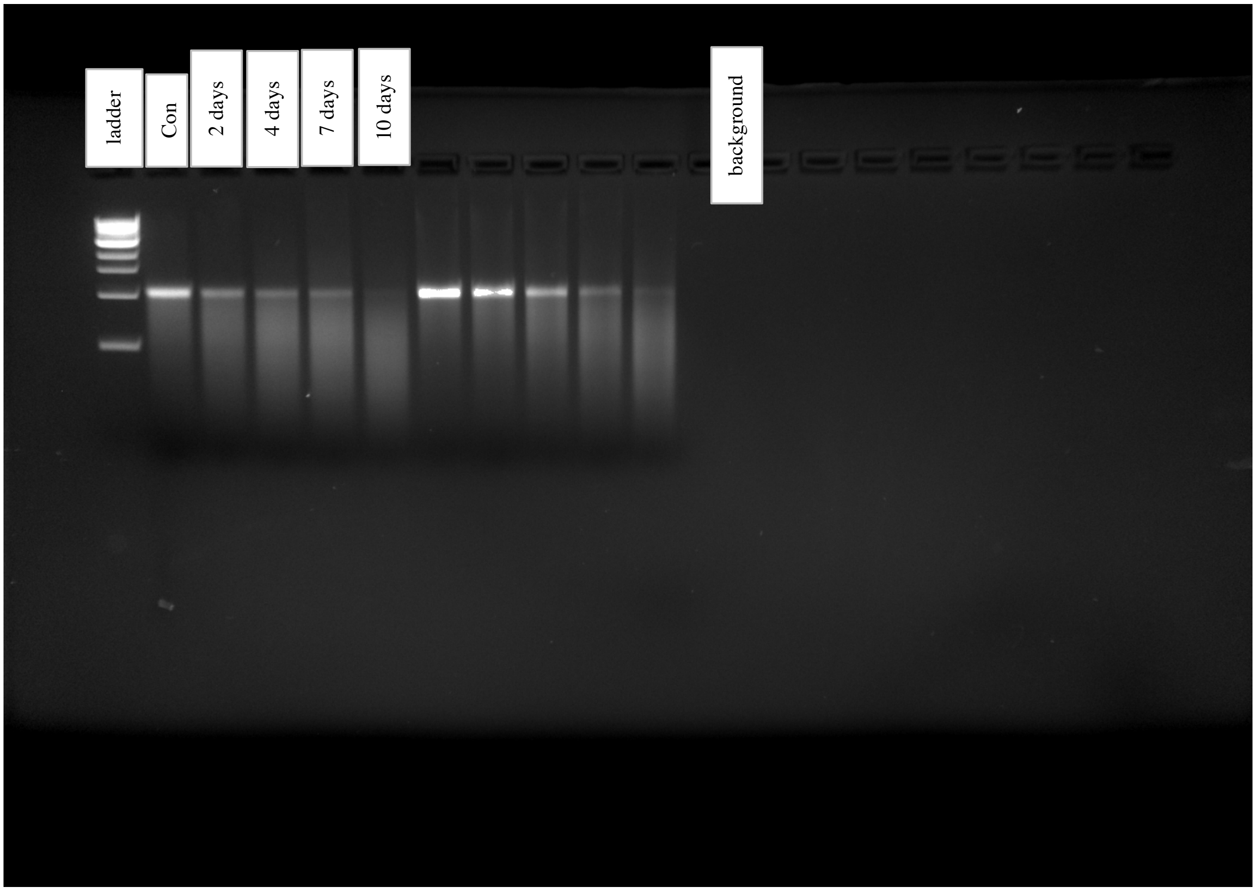

IVT_37_n3

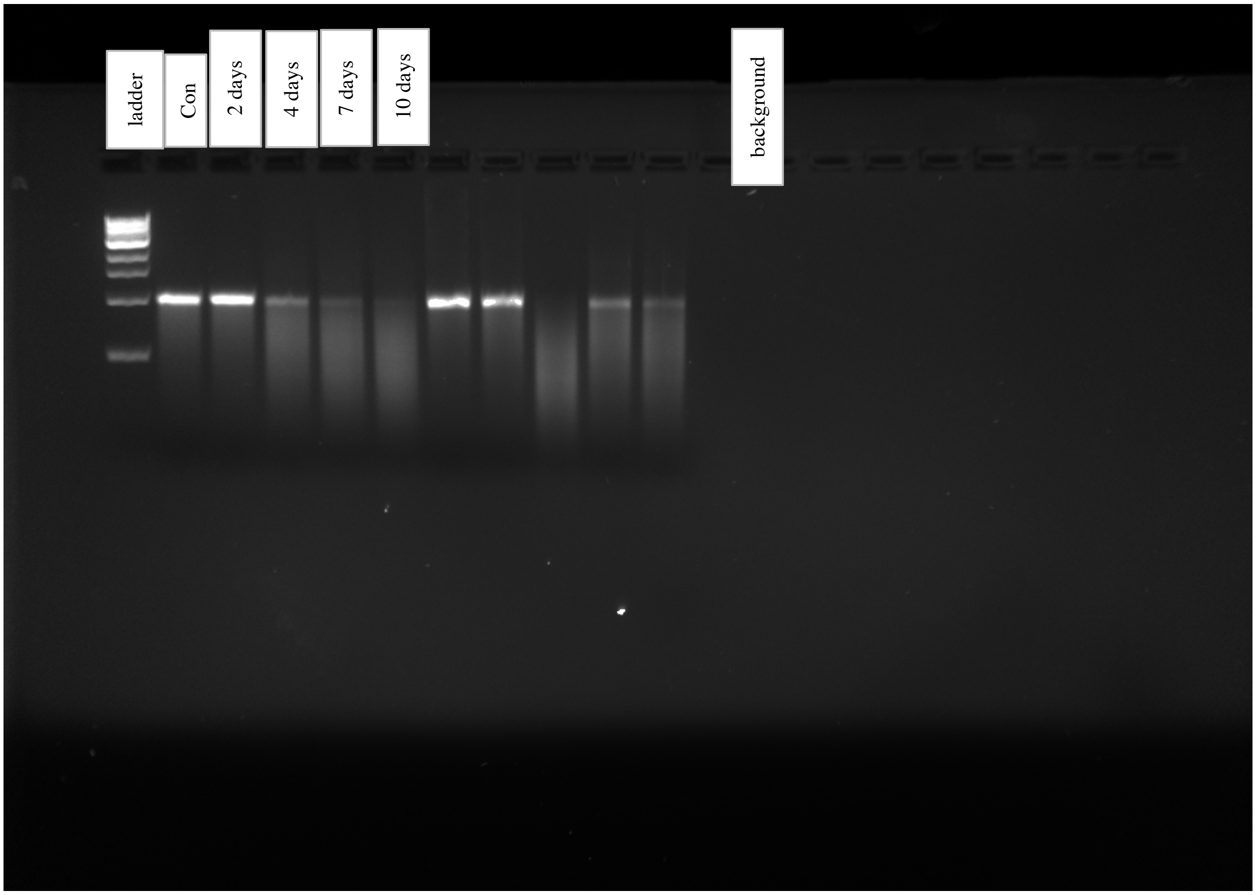

IVT_Bioanalyzer - star indicating the lanes used for the mauuscript

Assay Class: Eukaryote Total RNA Nano
Data Path: C:\...Eukaryote Total RNA Nano_DE20901499_2022-03-03_15-03-01.xad

Created: 2022-03-03 15:03:01
Modified: 2022-03-03 16:10:57

Gel Image

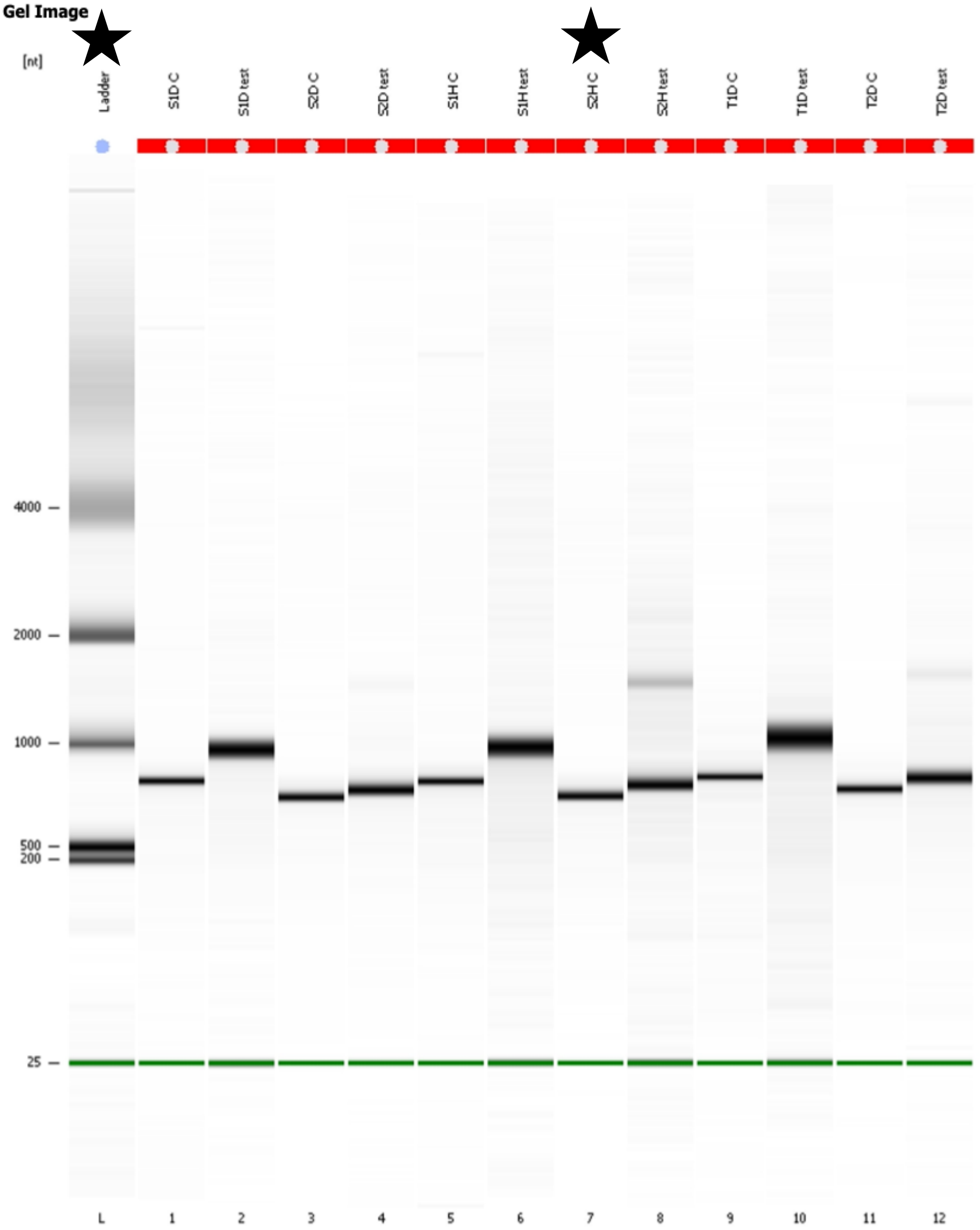

 © Copyright 2003 - 2009 Agilent Technologies, Inc.

IVT_Gel_corresponding_to_Bioanalyzer

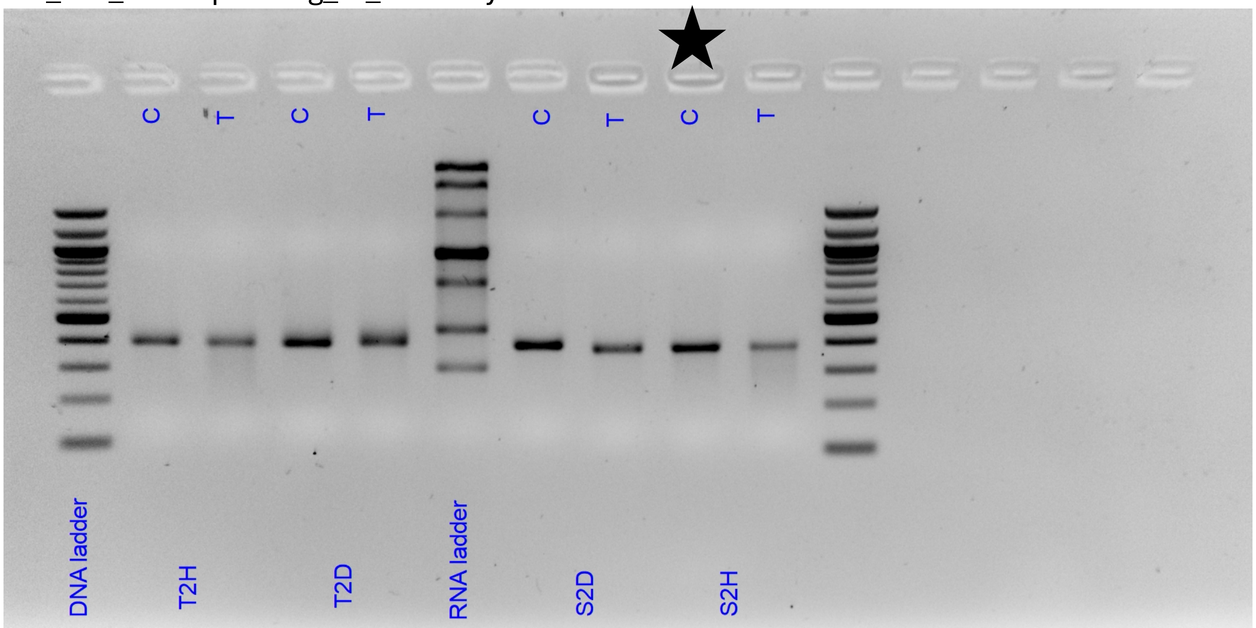

Tot_RNA__gel_37C_R1

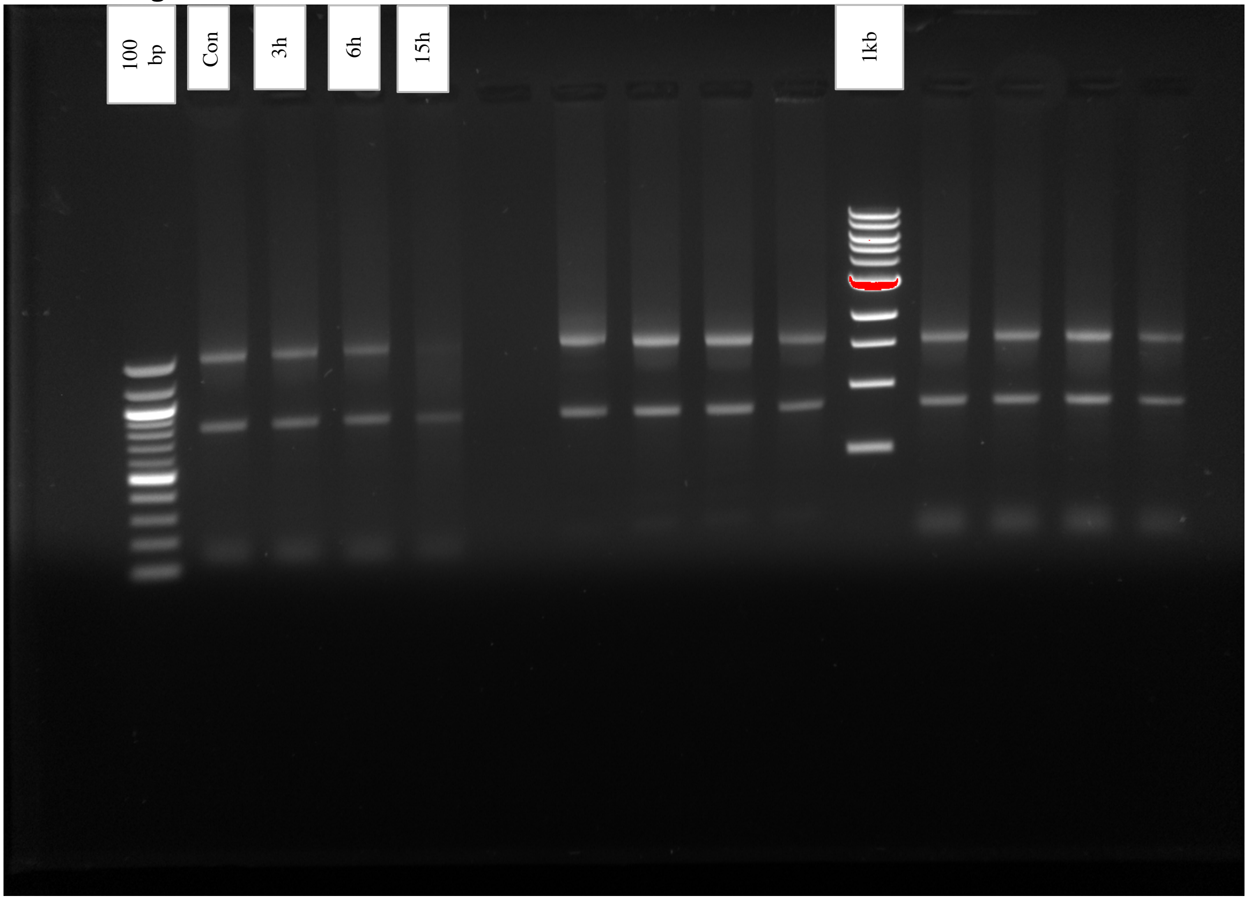

Tot_RNA_gel_37C_R2

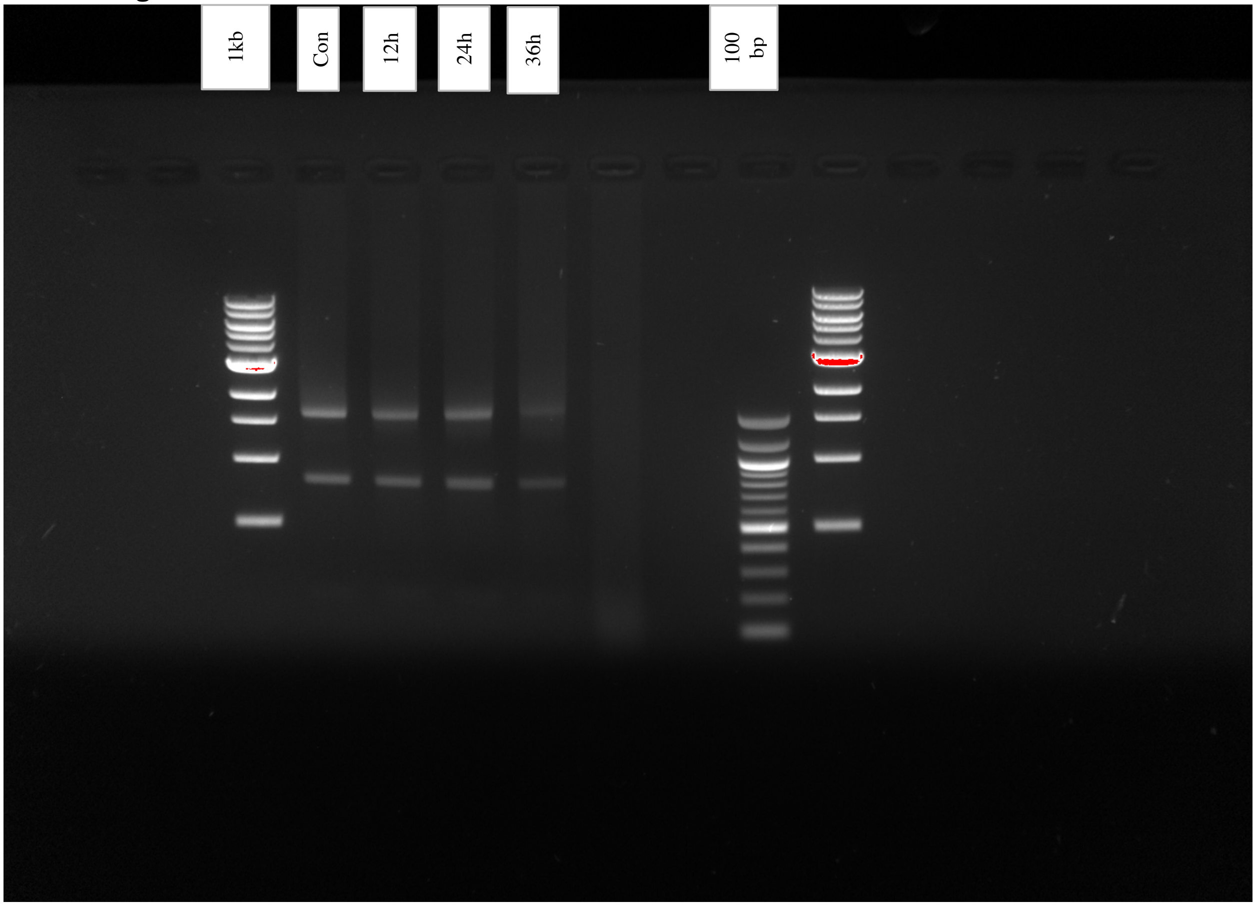

Tot_RNA_gel_45C_R3

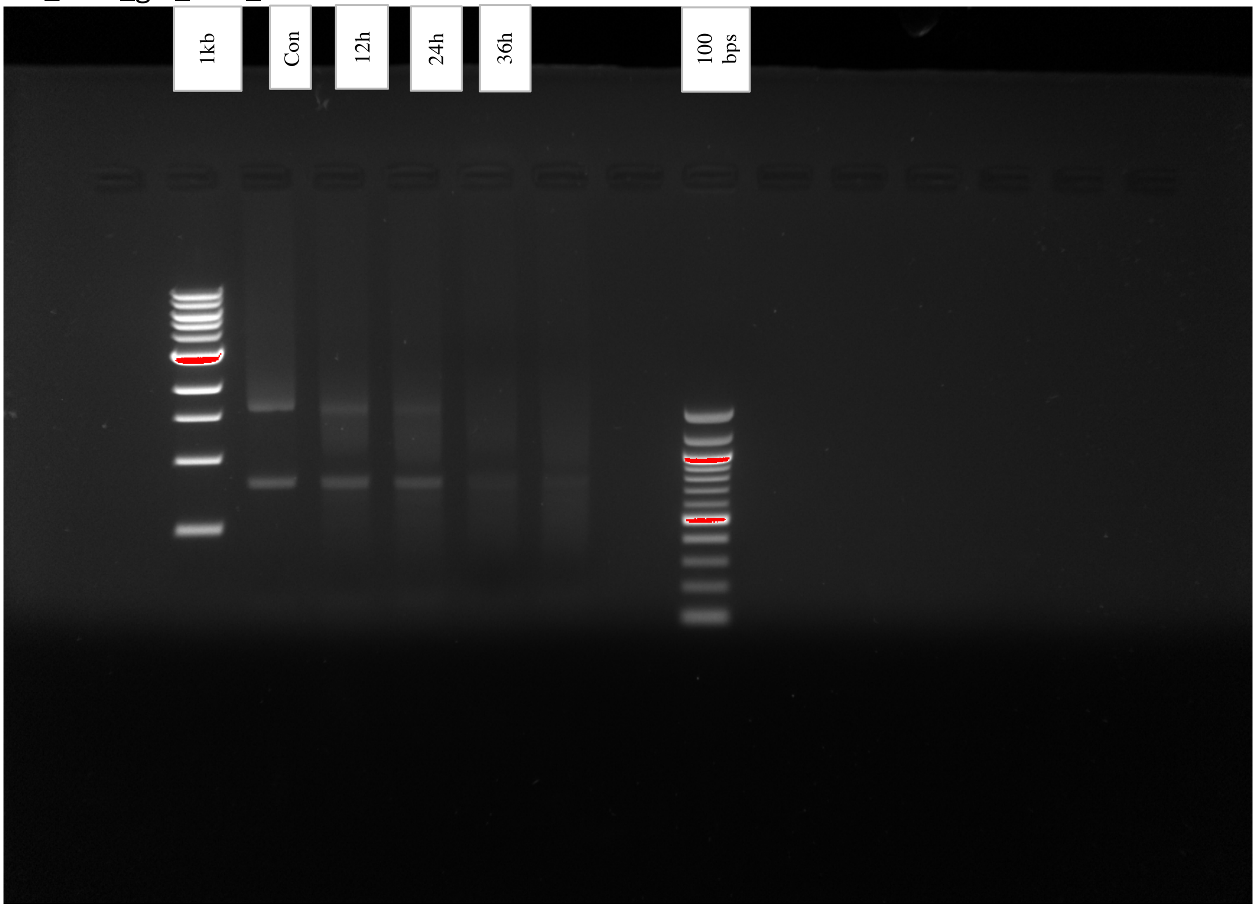

Tot_RNA_BA_37_R1

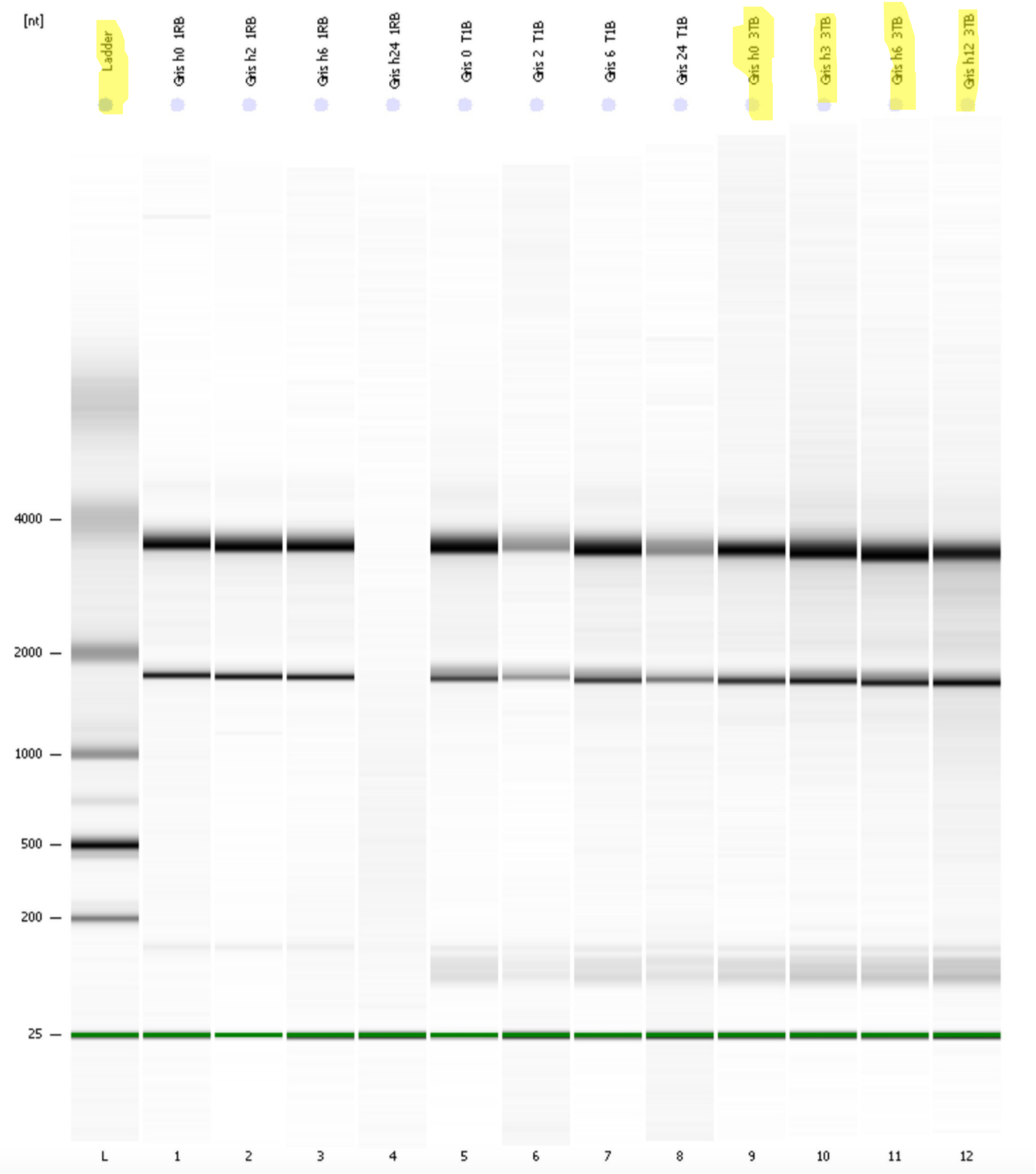

Tot_RNA_BA_37_R2

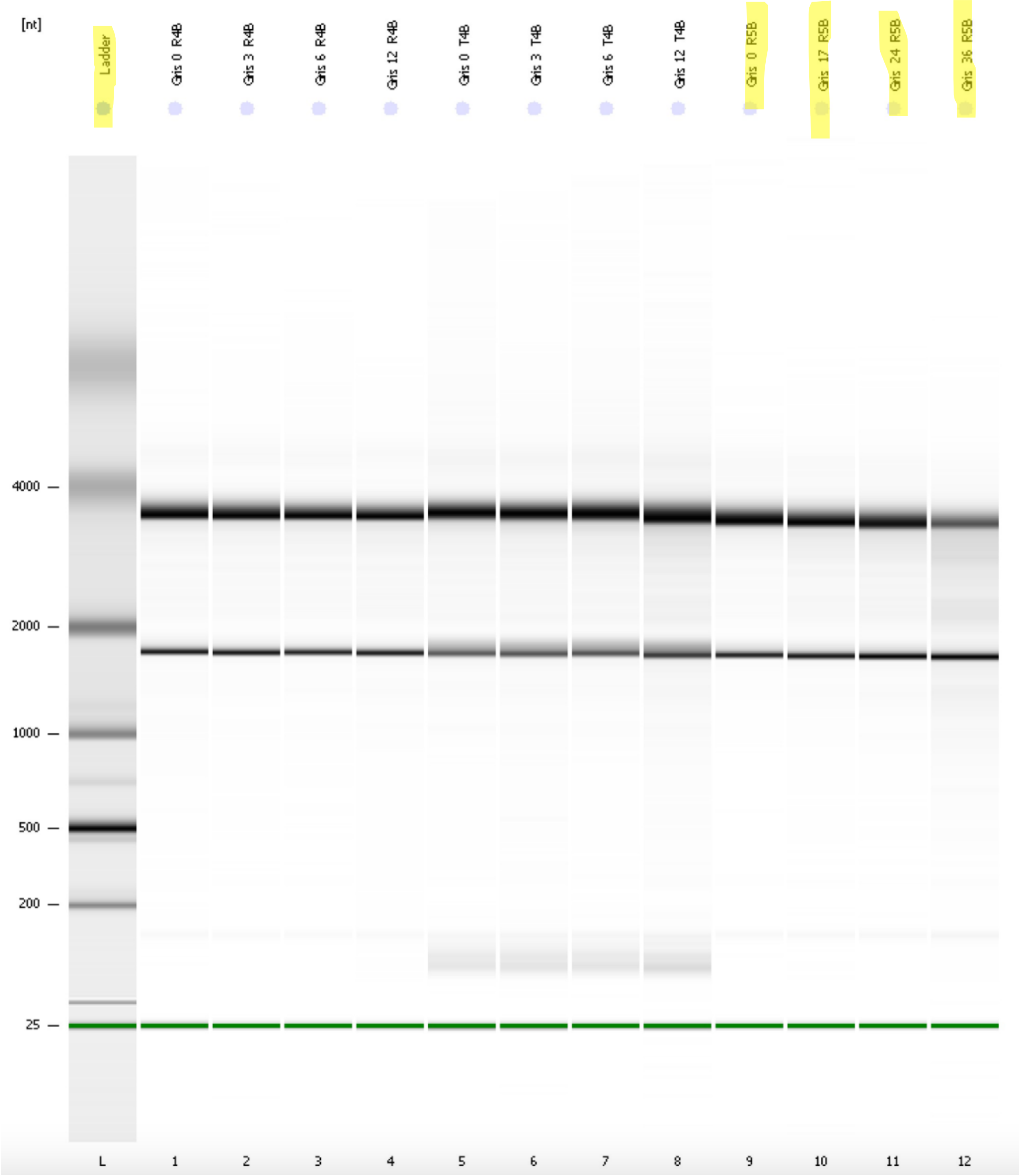

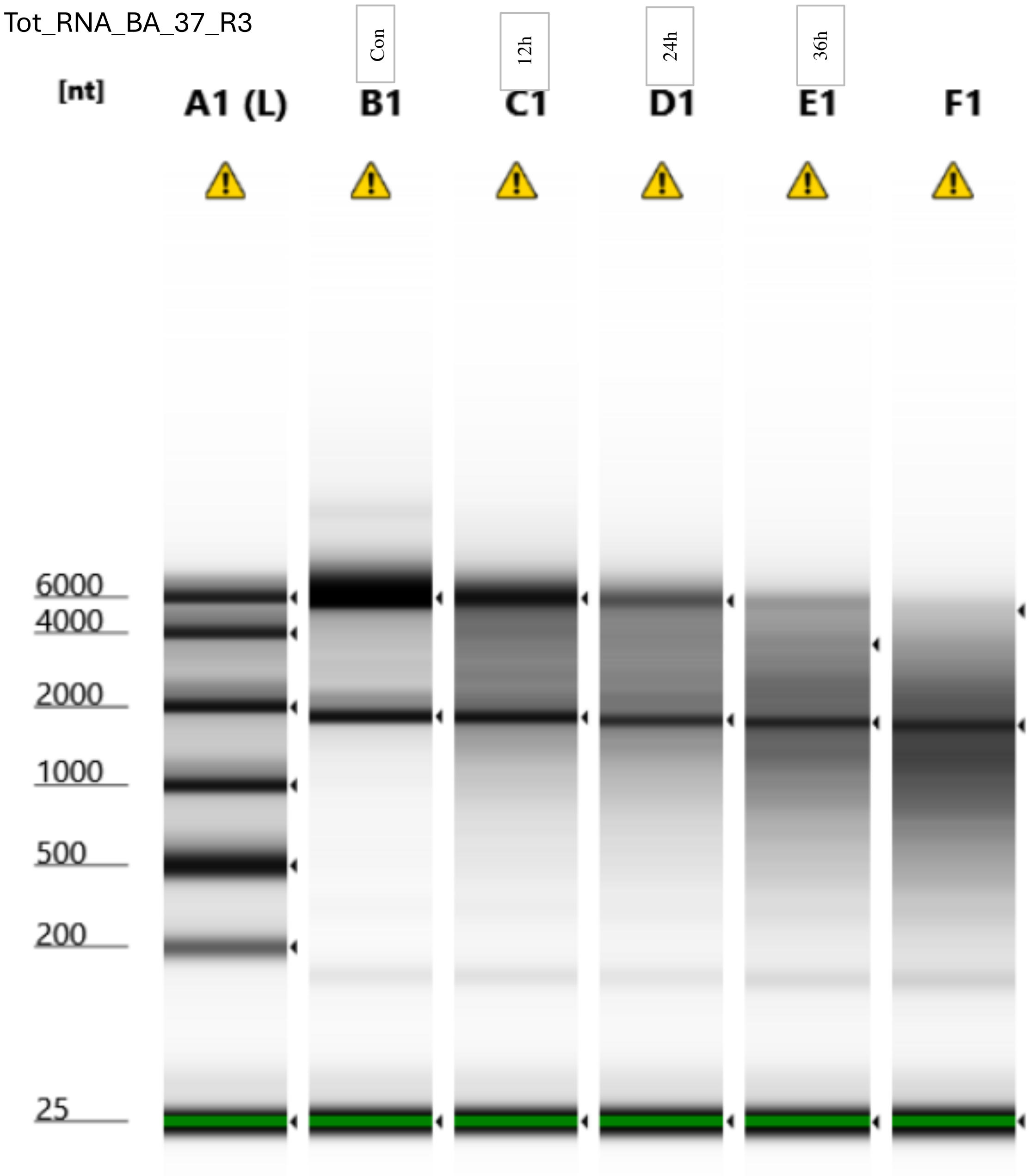
Tot_RNA_BA_37_R3
Con
12h
24h
36h
[nt]
A1 (L)
B1
C1
D1
E1
F1
6000
4000
2000
1000
500
200
25
